# Identifying the Activated Carbon Electrode Aging Pathways in Lithium-Ion Hybrid Capacitors

**DOI:** 10.1021/acsaem.4c01940

**Published:** 2025-01-10

**Authors:** Sylwia Slesinska, Bénédicte Réty, Camélia Matei-Ghimbeu, Krzysztof Fic, Jakub Menzel

**Affiliations:** † Institute of Chemistry and Technical Electrochemistry, Poznan University of Technology, Berdychowo 4, 60-965 Poznan, Poland; ‡ Institut de Science des Matériaux de Mulhouse (IS2M), Université de Haute-Alsace, CNRS UMR 7361, F-68100 Mulhouse, France; § Université de Strasbourg, F67081 Strasbourg, France; ∥ Réseau sur le Stockage Electrochimique de l’Energie (RS2E), CNRS FR3459, 80039 Amiens Cedex, France

**Keywords:** Li-ion capacitor, carbon electrode, organic electrolyte, aging mechanism, floating aging

## Abstract

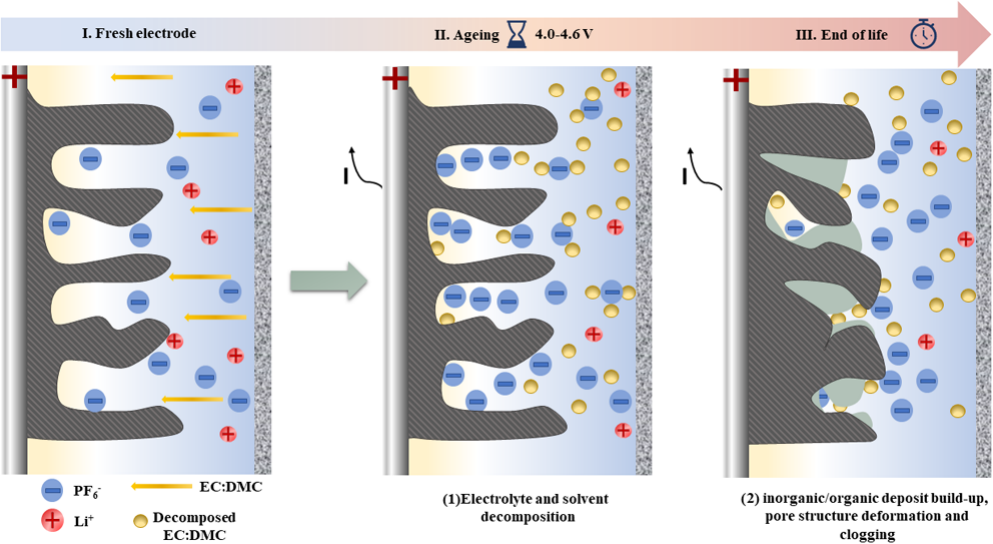

This paper reports on several mechanisms of carbon aging in a hybrid lithium-ion capacitor operating with 1 mol L^–1^ LiPF_6_ in an ethylene carbonate/dimethyl carbonate 1:1 vol/vol electrolyte. Carbon electrodes were subjected to a constant polarization protocol (i.e., floating) at various voltages and analyzed postmortem via several complementary techniques. The selected protocol was able to simulate the behavior of the real system. Due to the use of metallic lithium as the counter electrode, the influence of battery-like aging mechanisms was assumed to be limited. Our approach focused on the aging mechanisms related to the carbon electrode and determined the structural and chemical changes leading to energy fading in lithium-ion hybrid capacitors. It was shown that an increase in applied voltage not only results in faster system degradation but directs the aging chemistry to different pathways: at moderate voltage values, both capacitance loss and simultaneous increase in resistance predominate. This could be associated with the decrease in carbon surface area and possible pore clogging with by-products of electrolyte degradation and carbon oxidation disrupting the C sp^2^ network. When high voltage is applied, further oxidation of carbon occurs with an increase in measured resistance that leads to the relevant end-of-life criterion to be reached. Detailed postmortem analysis results attributed it to the formation of phenol and ether groups together with electrolyte decomposition products, higher oxidation levels, and structure degradation. It was evidenced that the decrease in the overall carbon conductivity and, in certain cases, modification of the textural properties ultimately aggravates the capacitor performance.

## 1 Introduction

From an industrial application point of view, porous carbon-based electrochemical capacitors (ECs) and Li-ion batteries (LiBs) are the most advanced technologies to date.^1−5^ The principles of operation of ECs and LiBs significantly vary. ECs utilize physical charge storage processes rather than chemical processes (as in LiBs); thus, the processes occurring are much faster and reversible, providing higher power density and much-improved cyclability (>10^6^ charge/discharge cycles).^6−8^ However, the EC’s energy density is much lower than that of LiBs. Therefore, the emergence of new technologies, such as hybrid capacitors (e.g., Li-ion capacitors (LiCs^9^)), can benefit from both systems, where the specific and volumetric energy densities can be significantly increased (12–15 Wh kg^–1^)^10,11^ while maintaining the high power and long-life characteristics of ECs.^12−14^

LiCs typically consist of one Li insertion material (e.g., Li_3_V_2_(PO_4_)_3_, Li_4_Ti_5_O_12_) and high surface area materials (e.g., activated carbon) as electrodes.^12,13^ To provide the long-term operation of these technologies, it is necessary to understand their degradation mechanisms. Although numerous studies have focused on the mechanisms of aging and proposed improvements in LiBs and ECs,^15−22^ limited work has been done in this area for LiCs, especially in terms of the aging of porous carbon electrodes. There is no doubt that the performance and longevity of LiCs are influenced by many factors: degree of prelithiation, operating temperature, applied electrode potential window, type of anode material, and instability of the lithiated anode, but their severity has been scarcely elaborated.^14,15,23−26^ Most research to date reports the growth of the solid electrolyte interphase (SEI) as the main aging mechanism in LiBs and LiCs, which can be addressed via an oversized negative electrode; however, this does not completely suppress it.^11,16^ Growth of the SEI on the surface of the negative electrode can increase its resistance, leading to a change in the potential of the positive electrode that can potentially generate LiF on the negative electrode above potentials of 4 V vs Li/Li^+^.^17^ Current aging studies on LiCs, e.g., when high voltage was applied with liquid and gel polymer electrolytes, reported degradation related to mechanisms occurring at the electrodes rather than those of the electrolytes.^27^ Other works report that the aging mechanisms strongly depend on the state of charge, especially concerning the negative electrode, where the growth of SEI was found to increase with the increase of the state of charge.^9^

Because LiCs combine LiB and EC electrode materials, aging can display similar characteristics to those found in electrochemical capacitors. In either an organic or an aqueous medium, this is related to the degradation of the positive electrode. In both cases, the measured Brunauer–Emmett–Teller (BET) specific surface area of the positive electrode after aging is significantly affected and reduced.^18−22,28−30^ Ultimately, the aging mechanisms of both electrodes would be complementary, and depending on the selected conditions and materials used, the aging mechanisms could vary and prove to be rather complex in nature. In that light, exploration of various experimental “scenarios” could bring about new knowledge, which seems promising for future optimization of these devices.

Carbon electrode aging can be achieved by applying different aging protocols, such as galvanostatic cycling or voltage holding tests, i.e., floating; these protocols are already widely described in the literature.^29,31,32^ Cycling stability tests are often evaluated by recording several thousands of galvanostatic charge–discharge cycles and are rather straightforward. Compared to floating tests (potentiostatic hold), they are, however, time-consuming. For example, in commercial electrochemical capacitors, 20% capacitance loss is observed after approximately 500 000–1 000 000 cycles.^33,34^ In comparison, when tested in potentiostatic mode, a device based on organic electrolyte (TEABF_4_/AN) suffered a 30% capacitance loss after 30 h and required 10 000 cycles during cycling tests to reach the same criterion.^32^ Additionally, it was shown that cycling and floating have varied influences on the time of operation, structure of the electrode, and resistance of the total cell in aqueous media.^29^ The former induces changes in the carbon structure with an insignificant influence on the measured resistance, whereas the former accelerates degradation. The latter is also termed “accelerated aging” due to the extended exposure time at high voltages in comparison to cycling; cycling is a reliable and time-efficient method of examining the state of health (SOH) of many energy storage devices, including LiC.^17−19^ To effectively monitor the process, both the loss of capacitance and the increase in equivalent series resistance (ESR) need to be simultaneously monitored during operation. According to the international standard (IEC 62391-1),^20^ a system failure is reported when the initial capacitance drops below 80% of its initial value or when the ESR has increased by 100%.

In this work, we report on the degradation mechanisms of porous carbon electrodes in LiCs at different elevated voltages: 4.0, 4.2, 4.4, and 4.6 V. The research was carried out with the application of a binder-free Kynol 507–20 activated carbon cloth as the working electrode and a typical LP-30 (1 mol L^–1^ LiPF_6_ in an ethylene carbonate/dimethyl carbonate 1:1 vol/vol) electrolyte designed for Li-ion batteries. The proposed half-cell configuration allowed the isolation of the fundamental processes occurring at the carbon electrode only. This eliminated the complexities introduced by a full cell. Thus, a detailed study of the aging mechanisms associated with that specific electrode was achieved. The combined information gathered from postmortem analyses, including elemental analysis, Raman spectroscopy, temperature-programmed desorption mass spectrometry (TPD-MS), X-ray photoelectron spectroscopy (XPS), and porosimetry, elucidated the degradation paths and microstructural changes of the electrodes.

## 2 Experimental Section

### 2.1 Electrodes and Electrochemical Cell Preparation

Circular self-standing electrodes (Ø = 16 mm, 18.6 mg) were cut from activated carbon fabric (Kynol ACC 507-20, Germany). Before electrochemical measurements, the carbon material was dried at 120 °C for 12 h under a vacuum to eliminate water and avoid potential oxidation. After electrochemical aging, the electrodes were washed in water and ethanol to remove the soluble impurities and then dried at 120 °C for 12 h under vacuum.

A solution of LiPF_6_ salt at a concentration of 1 mol L^–1^ dissolved in 1:1 vol EC/DMC was purchased from Merck, Germany. The water content in the electrolyte declared by the producer was less than 20 ppm.

Electrochemical measurements were made on two-electrode ECC-ref cells (El-Cell, Germany). Before measurement, the electrode was soaked in the electrolyte and separated by a GF-D porous membrane (Whatman, UK). Metallic lithium was used as the counter electrode.

### 2.2 Electrochemical Analysis

Electrochemical measurements were performed with a computer-controlled multichannel potentiostat/galvanostat (VMP3, Biologic, France). Experimental techniques included galvanostatic charge/discharge with a current load of 0.5 A g^–1^, cyclic voltammetry at scan rates of 2 mV s^–1^, and determination of the current-interrupted ohmic drop at a current load of 0.2 A g^–1^ for 0.05 s. The floating protocol consisted of a 2 h voltage hold at terminal voltages interrupted by capacitance and resistivity measurements. All current and capacitance values were expressed per mass of one electrode if not stated otherwise.

### 2.3 Porosity Analysis

The nitrogen adsorption/desorption isotherm of the activated carbon electrode before and after aging was recorded using an ASAP 2460 analyzer (Micromeritics, USA) at 77 K. Prior to analysis, the samples were purged under helium flow for 24 h at 120 °C and then placed under high vacuum for 5 h. The low temperature of degassing was selected in order to avoid surface modification of carbon while ensuring the physisorbed species.^35^ The specific surface area was calculated using the BET equation and verified with the pore size distribution, calculated using the 2D Non-Local Density Functional Theory (2D NLDFT) method.^36,37^

### 2.4 Elemental Analysis

The determination of the mass fractions of carbon, hydrogen, nitrogen, and oxygen was carried out with an Thermo Scientific FlashSmart equipment. The amount of oxygen was obtained through a direct (separate) elemental analysis. Our results are the averages of three separate analyses.

### 2.5 XPS Analysis

X-ray photoelectron spectroscopy (XPS) was performed with a VG SCIENTA SES-2002 spectrometer equipped with a concentric hemispherical analyzer. The incident radiation was generated by a monochromatic Al Kα X-ray source (1486.6 eV) that operated at 420 W (14 kV; 30 mA). A wide scan (survey) spectrum was recorded with a pass energy of 500 eV, and for high-resolution spectra, the pass energy used was 100 eV. The spectra were subjected to a Shirley baseline, and peak fitting was performed with mixed Gaussian–Lorentzian components with equal full width at half-maximum (fwhm) using CASAXPS version 2.3.18 software. All binding energies (BEs) are referenced to the C 1s (graphite-like sp^2^ carbon) peak at 284.5 eV and given with a precision of 0.1 eV.

### 2.6 TPD-MS Examination

Measurements were carried out using a home-built TPD-MS setup under a secondary vacuum (10^–5^ Pa).^21,38^ This equipment included an Inficon Transpector 2 mass spectrometer and a Leybold ITR100 Bayard-Alpert ionization vacuum gauge, which recorded continuous measurements of the intensity of the masses (*m*/*z*) of the released gases and the total gas pressure while the material was heated at a constant temperature rate. Before material analysis, the TPD-MS system was calibrated by separately measuring the mass spectrometer intensity and pressure of each gas: CO_2_ (*m*/*z* = 44), CO (*m*/*z* = 28), H_2_O (*m*/*z* = 18), and H_2_ (*m*/*z* = 2). When the mass intensities and pressure measured during material analysis were correlated with the calibration results, the desorption rate (μmol s^–1^ g^–1^ of material) of each gas could be determined versus the temperature. Moreover, the time integration of the TPD-MS curves provided the total amount of each gas per gram of material. In this study, TPD-MS measurements were performed under the following experimental conditions. The sample was placed in a quartz tube and heated to 950 °C at 10 °C/min. The maximum temperature was then maintained for 30 min before cooling. From the measured mass intensities and the calibration data, the pressure was calculated based only on the calibrated gases. By comparison of this calculated pressure with the real pressure measured by the vacuum gauge, the presence of uncalibrated gases was determined.

### 2.7 Raman Spectroscopy

Raman spectroscopy was performed with a DXR-2 computer-controlled Raman microscope (ThermoFisher Scientific, USA). The electrode spectrum was collected with the application of a 532 nm laser with an adjusted power of 8 mW. Analysis of the data obtained was performed with the Origin2021 software application. The peak positions and integration of the D/G bands were determined by deconvolution of the experimental spectrum.

## 3 Results and Discussion

**Figure 1:**
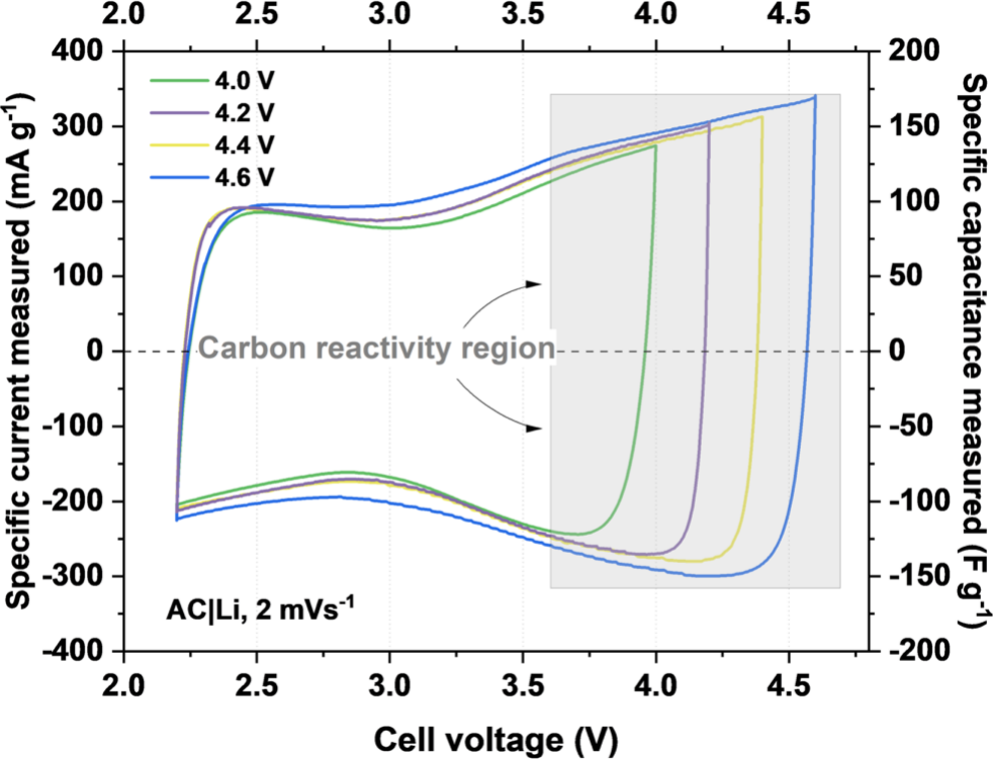
Cyclic voltammetry profiles (2 mV s^–1^) of the Kynol ACC 507-20/metallic lithium system with 1 mol L^–1^ LiPF_6_ in EC/DMC as the electrolyte; all scans start from 2.2 V, and terminal voltages were set to 4.0, 4.2, 4.4, and 4.6 V.

Figure 1 shows a cyclic voltammetry (CV) profile with a gradual voltage increase cutoff point from 4.0 to 4.6 V.

The voltammograms showed a characteristic “butterfly” shape, typical of LiC and organic-based EC, originating from different charge storage mechanisms and potential profiles for the electrodes. No sharp current increases, even at high voltage values, are considered as the lack of typical signs of system degradation; however, the interfacial activity of carbon should not be excluded. More detailed analysis indicates that the charge is symmetrically distributed with nearly 100% retention, also suggesting a lack of side reactions. It has been therefore assumed that 4.6 V is still the safe operating voltage, and the long-term performance was tested via potentiostatic hold, where the end-of-life criterion was defined as 20% of the initial capacitance loss or 100% resistance increase according to the international standard (IEC 62391-1).^20^

**Figure 2:**
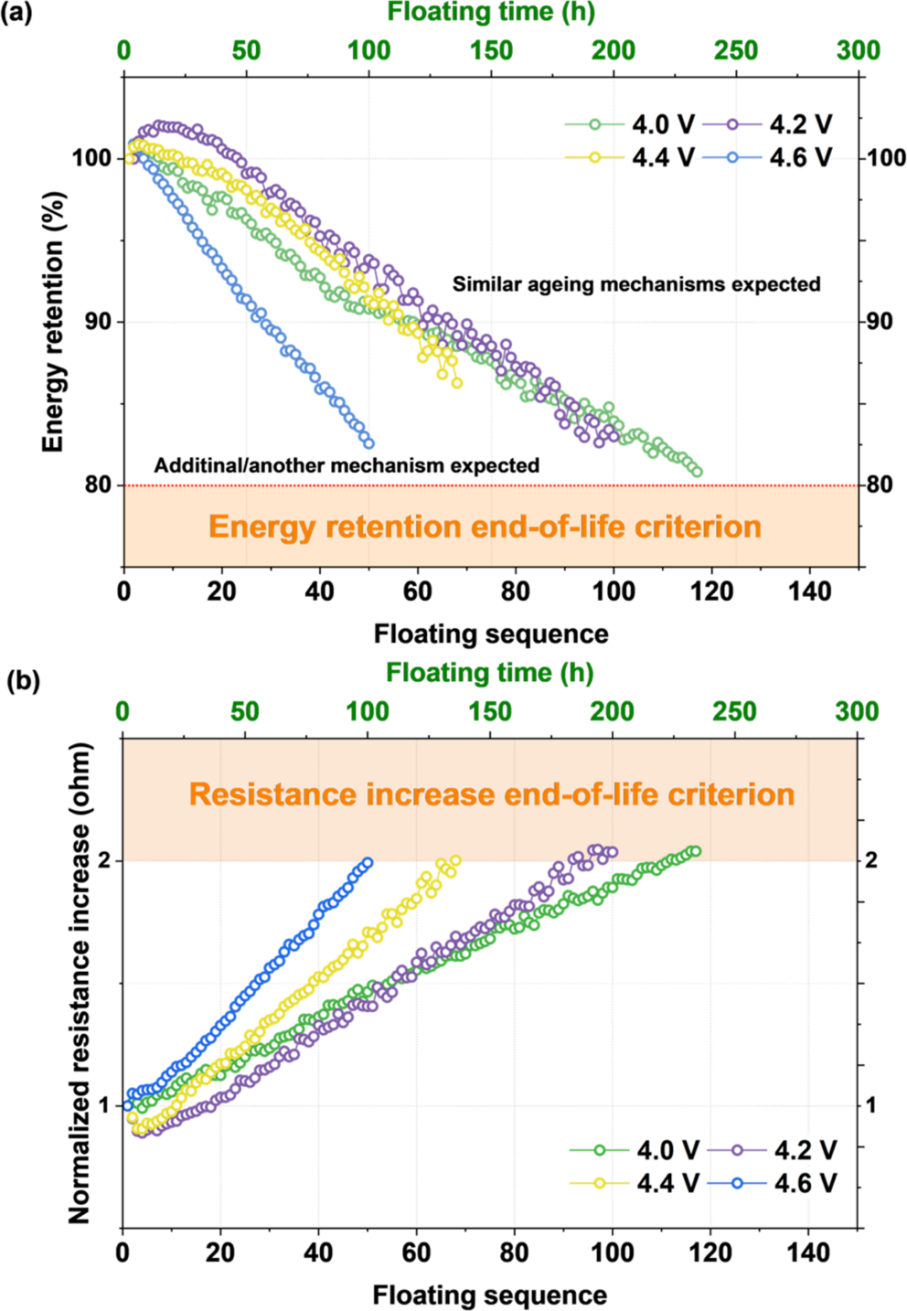
Results of the floating test for the LiCs with a 1 mol L^–1^ LiPF_6_ electrolyte at various voltages (4.0–4.6 V). (a) Energy retention vs floating time and (b) relative resistance vs floating time.

Figure 2 shows the changes in energy profiles with floating time and the corresponding increase in resistance with floating time at a given voltage. Additional charge/discharge curves before and after floating can be found in Figure S1.

From these data (Figure 2 and Table 1), two characteristic aging behaviors that significantly vary with the change in applied voltage are distinguished. At the voltage of 4.0 V, the loss of energy and the increase in resistance follow a similar linear trend, and the cell with the highest durability among all systems studied is achieved, where the resistance end-of-life criterion and capacitance loss are reached after ∼240 h. Furthermore, there is a significant change when a higher voltage is applied. At 4.2 V and above, the resistance end-of-life criterium is reached while capacitance retention is above 80%. Moreover, with an increase in the voltage, the lifetime of the cells is limited from ∼200 h for 4.2 V to ∼100 h for 4.6 V voltage limitation. Additionally, the symptoms of electrolyte decomposition in initial cycles are observed in the form of decreased measured cell resistance, resulting from an increase in internal pressure, as evidenced by operando pressure measurements at 4 V (Figure S2). Importantly, these results indicate that when considering accelerated aging experiments, it is crucial to monitor capacitance, energy retention, and resistance parameters since they do not necessarily change in an equal manner, especially when voltages close to or beyond the stability limit are concerned.

**Table 1:** Comparison of Energy, Capacitance Retention, Resistance Increase, and End of Lifetime after Reaching the End-of-life Criterion

cell voltage (V)	4.0	4.2	4.4	4.6
energy retention (%)	83	84	84	83
capacitance retention (%)	80	82	86	86
resistance increase (%)	100	100	100	100
end-of-lifetime (h)	234	200	136	100

**Figure 3:**
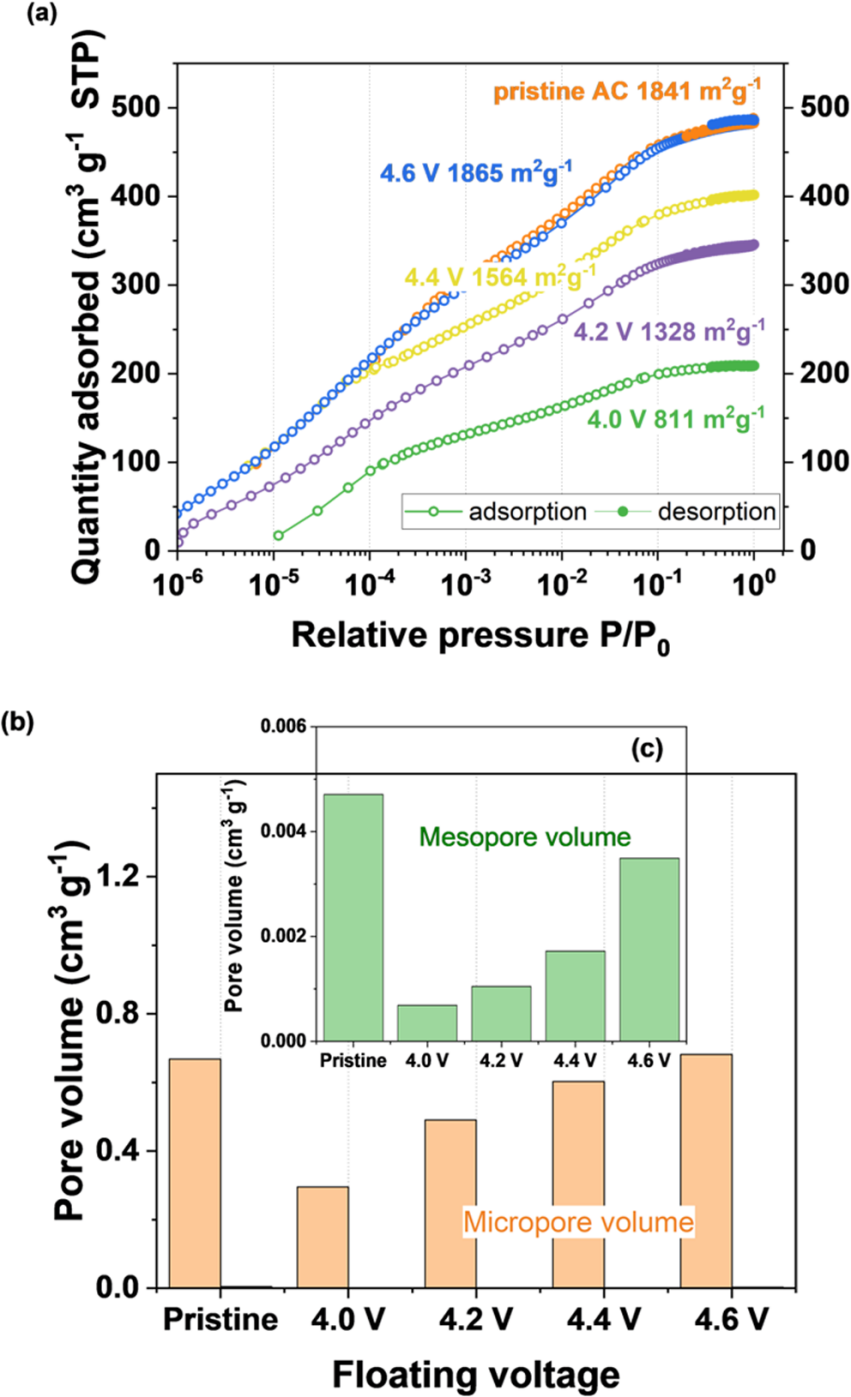
Porosity measurements for the pristine carbon cloth and the positive electrodes after the floating test at various voltages (4.0–4.6 V), (a) N_2_ isotherms at 77 K, (b) comparison of *V*_micro_ and *V*_meso_ from 2D NLDFT, and (c) inset showing the *V*_meso_.

To further explain the aging and pore clogging phenomena, a series of postmortem analyses were completed to determine the electrochemical changes with any chemical or structural differences that occurred in the carbon electrodes and compared with the pristine Kynol 507-20 material, as the aging mechanism is not possible to deduce solely from the electrochemical data. Figure 3a shows N_2_ adsorption/desorption isotherms measured at 77 K. To better demonstrate the changes in the micropore-related relative pressure regions, the *x*-axis is presented on a log scale. Pore size distribution is presented in Figure S3.

The material is characterized by a type I isotherm and a microporous texture with an additional mesopore contribution; the BET and 2D NLDFT surface area was ca. 1800 m^2^ g^–1^. The results showed a significant decrease in the specific surface area of the electrodes floated at 4.0 V, where the value changed from 1841 to 811 m^2^ g^–1^. Moreover, *V*_micro_ decreased from 0.67 cm^3^ g^–1^ for pristine carbon to 0.29 cm^3^ g^–1^ for the positive electrode aged at 4.0 V. In addition, *V*_meso_ decreased as well, approaching values close to zero. This followed the trend for water-based systems in which pore clogging by solid precipitates and residuals was observed.^28^ It has to be mentioned that the profile of the isotherms suggests that the order of pore blocking is similar for both voltages; however, the severity (reflected by the quantity of adsorbed gas and relevant surface area decrease) changes with the voltage. This might also suggest that there is a significant role of the polarization protocol and pore accessibility. Additionally, the different nature of by-products of electrolyte decomposition being potential dependent might result in pore clogging at 4.0 V.^39^ Noteworthy, for higher floating voltages (4.2 and 4.4 V), the change in *S*_BET_ was much less pronounced, with a decrease to 1328 and 1564 m^2^ g^–1^, respectively. The volume of pores appeared to be less affected as the time necessary to reach the end-of-life criterium decreased. At 4.6 V, *S*_BET_ and *V*_micro_ remained unchanged, where only a decrease in *V*_meso_ was observed. These results were strongly correlated with the electrochemical results, where the two aging pathways were considered. As aforementioned, the *S*_BET_ degradation might be connected with the time of the experiment where for the system aged at 4.0 V, more mechanical stress was applied than in the case of the system aged at >4.2 V. To better understand the involved processes, an elemental analysis of pristine and aged electrodes was performed (Figure S4a). This helped to quantify the elemental composition after floating at various voltages. Compared to the pristine electrode, a significant increase in oxygen content was found, i.e., from 1.5 to 5.0%, at 4.0 V. For higher voltages, a slight increase was further observed (7%), which remained rather constant for the electrodes cycled between 4.2 and 4.6 V. This implied the role of electrode oxidation on carbon conductivity decrease, which did not exponentially increase with a higher floating voltage but rather slightly changed by ∼2% of oxygen. The same results were confirmed via XPS data analysis (Figure S4(b) and Table 2), where the surface composition of the electrodes was determined. In comparison to the pristine material, all electrodes displayed an increase in the oxygen content, with a simultaneous decrease in the carbon content. Traces of phosphorus were also present in all samples and increased by ∼0.1% with higher voltages (Table 2).

**Table 2:** XPS Chemical Composition of the Aged Electrodes at 4.0 and 4.6 V

element	C 1s	O 1s	N 1s	P 2p	F 1s	S 2p
pristine	92.01	5.53	0.00	0.16	0.00	0.00
4.0 V	81.12	17.54	0.00	0.23	0.00	0.00
4.6 V	78.64	19.92	0.54	0.34	0.26	0.26

At 4.6 V, 0.26% fluorine was found, indicating the decomposition of the LiPF_6_ salt; this result is discussed in more detail in the context of TPD-MS analysis data. Therefore, the oxidation of carbon and formation of new, inorganic, and organic species with voltage was confirmed. A progressive decrease in the carbon content on the surface was also observed, but it is rather the result of increased content of other elements on the electrode surface due to electrolyte decomposition. However, the formation of gaseous products and degradation of the electrode cannot be excluded either. At initial cycles at high voltages, the oxygen functional groups found on the surface of the carbon cloth may be eventually oxidized to CO_2_ and H_2_O.^40^ According to the literature, this could lead to the formation of Li_2_O and H_2_ on the negative electrode, which could deplete cyclable Li^+^ ions and produce gas and HF.^41^ However, as XPS is a surface technique, the exact state of the carbon electrode underneath the solvent/electrolyte degradation compounds cannot be assessed. Ar-etching experiments would be useful to gain insight into the chemical composition at different penetration depths of the electrode.

**Figure 4:**
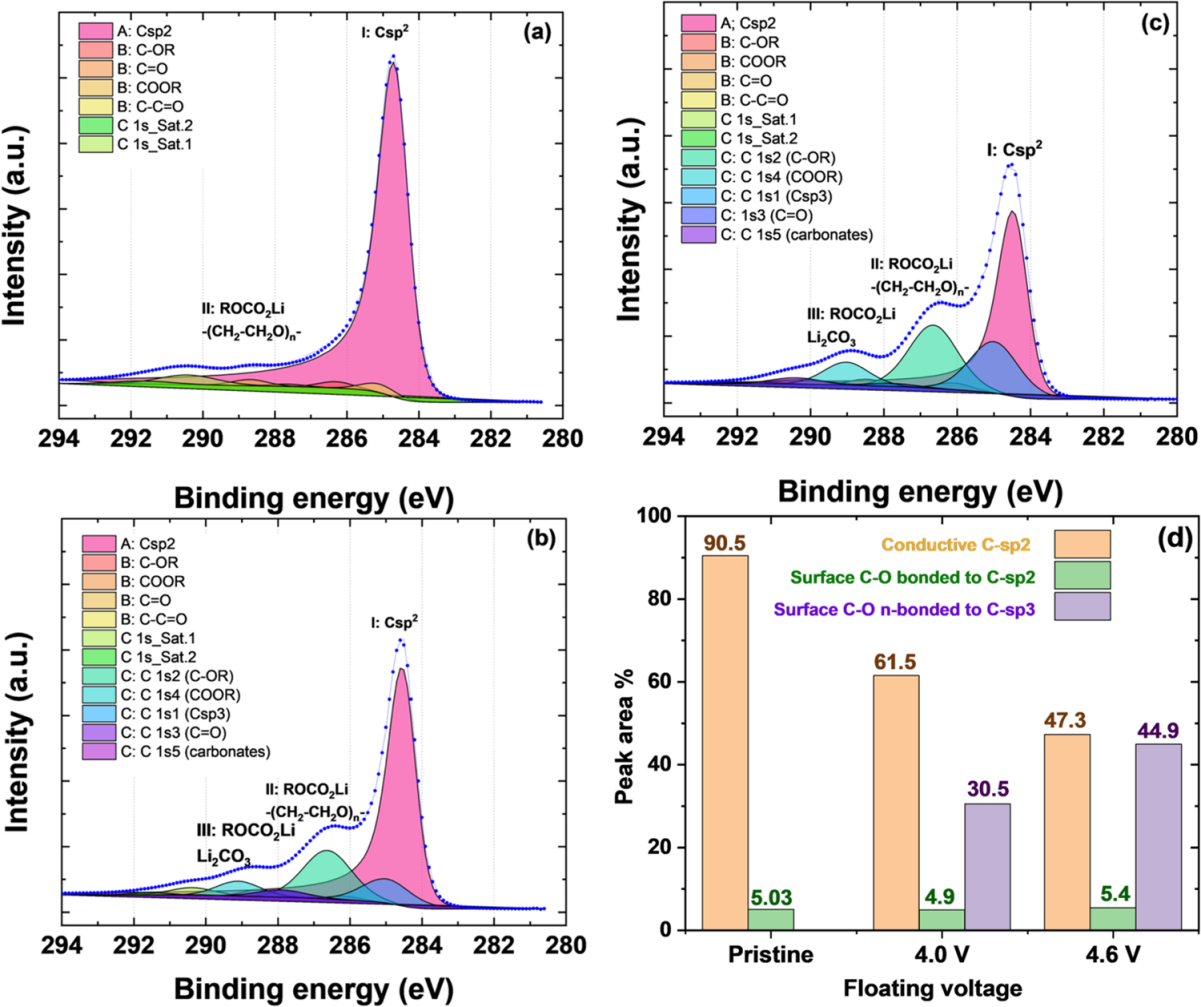
Deconvoluted XPS C 1s spectra for (a) pristine carbon; (b) carbon electrode after aging at 4.0 V; (c) carbon electrode after aging at 4.6 V; (d) surface compositions of the carbon electrodes prior to and after the floating test at different voltages. Color code: orange corresponds to C sp^2^, green corresponds to C–OR, COOR, C=O, and C–C=O bonded to C sp^2^, and violet corresponds to C–OR, COOR, C=O, C sp^3^, and carbonate fractions.

Moreover, the oxygen content obtained from XPS was higher than that from the elemental analysis, which further indicated that the aging process occurred mostly at the electrode/electrolyte interface. The fast oxidation of the carbon surface leading to a resistivity increase might explain the short lifespan and less affected *S*_BET_ as the terminal voltage increases. In addition, the detailed deconvolution of carbon XPS spectra is shown for the aged electrodes prior to and after the aging procedure at 4.0 and 4.6 V (Figures 4 and S5).

The fraction of conductive C sp^2^ significantly declined with floating voltage, from 61.5 to 47.3%, and carbon oxidation led to a decrease in conductivity. The presence of C–O functional groups bonded to C sp^2^ remained rather constant (∼5%), while those not bonded to C sp^2^ significantly increased in comparison to the pristine carbon cloth. The lack of this component on the pristine carbon, its later appearance at 4.0 V, and its notable increase at 4.6 V indicated electrolyte/solvent decomposition.

The C component corresponded to species, such as lithium alkyl carbonate (ROCO_2_Li), lithium carbonate (Li_2_CO_3_), and poly(ethylene oxide) polymer (CH_2_–CH_2_–O), formed on the electrode surface via the polymerization of EC already described in the literature.^41,42^ The presence of these species was observed on the C 1s peaks at 286.48 eV (CH_2_–CH_2_–O) and 288.95 eV (LiCO_3_). ROCO_2_Li had a doublet at both indicated binding energies, corresponding to the CO-like (286.48 eV) and CO_3_-like (288.95 eV) carbons. The solvent decomposition leads to lithium methyl carbonate (LiOCO_2_CH_3_) and lithium ethyl dicarbonate ((CH_2_OCO_2_Li)_2_).^43^ These compounds were usually observed in the SEI formed on graphite materials cycled in the same electrolyte as used in the present study.^44^ Their role is different, and the proportion of each species in the SEI layer impacts its properties. For example, Li_2_CO_3_ formed by the reaction of lithium with the electrolyte and/or solvent protects the carbon surface from further electrolyte decomposition. The organic alkyl carbonates derived from the solvent decomposition present an insulating nature, affecting the electrode conductivity. Nevertheless, the polymeric species (PEO) formed by carbon surface interaction with the electrolyte provide mechanical integrity to the SEI to withstand volume changes and stress occurring during cycling process.^45^ However, the nature of these polymeric species can be different depending on the potential. It was demonstrated that at high voltages (>4.2 V vs Li^+^/Li) the PEO might undergo oxidative polymerization via radicals (-O.), forming longer PEO chains and various gases.^46^ Therefore, these longer-chain polymers formed at high voltage might rather block the mesopores, while the small-chain polymers formed at low voltage (4.0 V) are more susceptible to deposition in the micropore, blocking the entries and leading to a decrease of *S*_BET_ and micropore volume.

As both inorganic and organic species are found in the SEI, a compromise between electronic insulation and ion conductivity is ensured.

**Figure 5:**
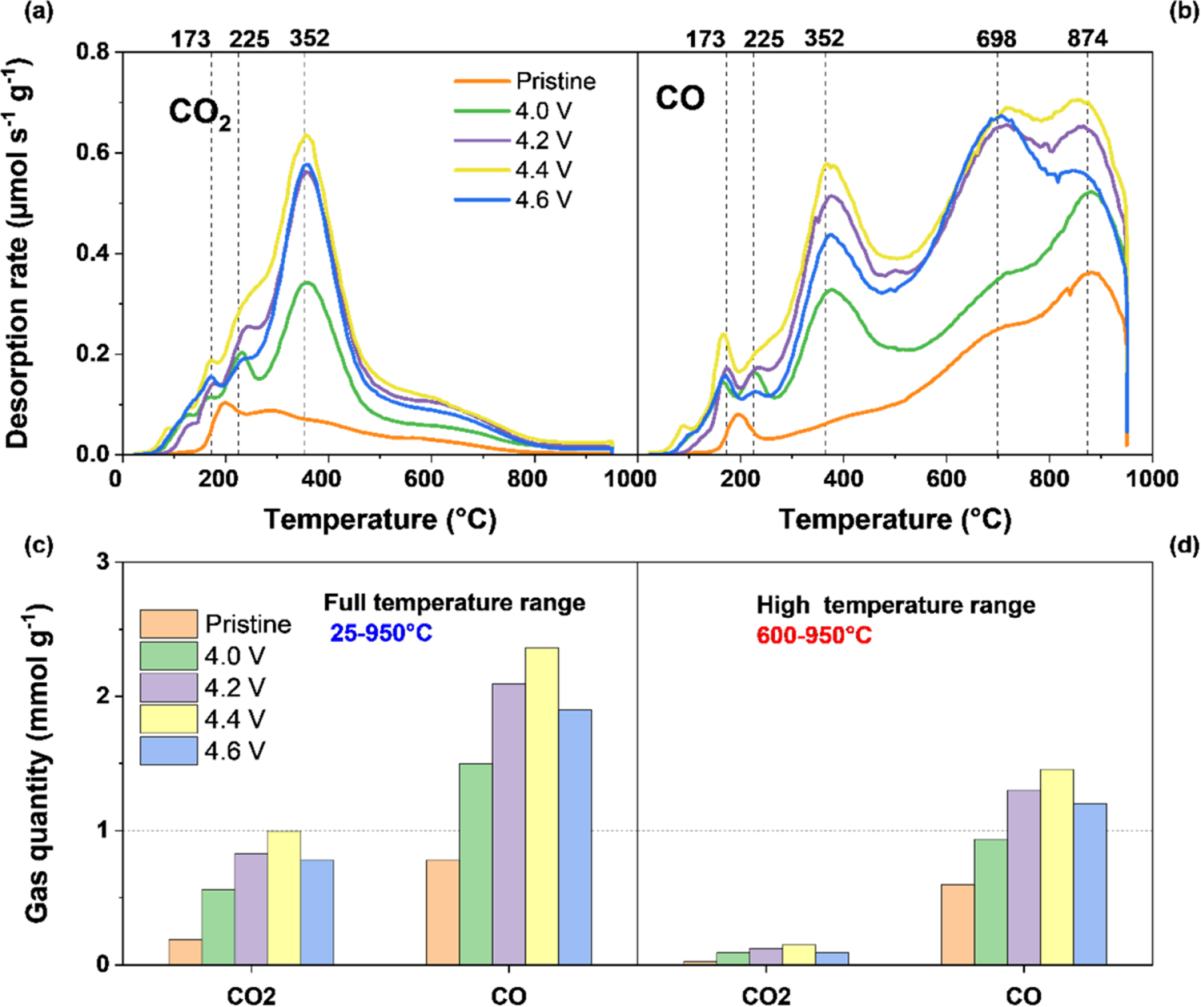
TPD-MS profiles of the pristine and cycled electrodes: desorption rates of (a) CO_2_ and (b) CO and the total gas quantities (c) between 25 and 950 °C and (d) between 600 and 950 °C.

The formation of all of these species confirmed the presence of an insulating layer on the carbon surface. This layer impeded the observation of new oxygen-based functional groups formed on the carbon surface after cycling, considering the low penetration depth of the XPS technique (maximum 10 nm). However, the TPD-MS results, which enabled bulk analysis of electrodes (Figure 5c,d), showed both the formation of oxygen functional groups and electrolyte degradation products.

**Figure 6:**
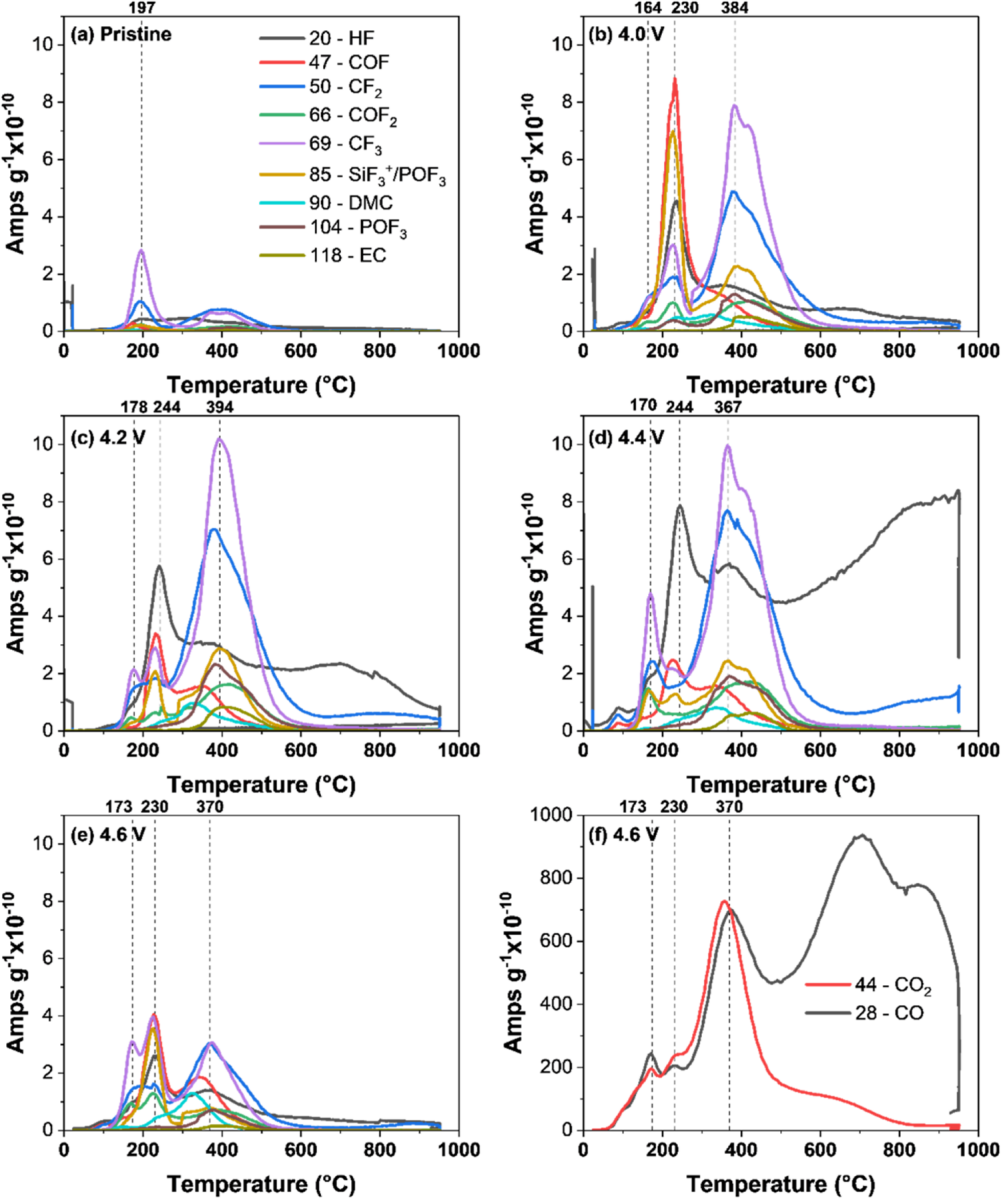
MS profiles for uncalibrated gases from thermal decomposition of (a) pristine material, (b) 4.0 V, (c) 4.2 V, (d) 4.4 V, and (e) 4.6 V. (f) MS profiles for the masses related to CO and CO_2_.

Temperature-programmed desorption coupled to mass spectrometry (TPD-MS) is a qualitative and quantitative measurement that is used to characterize the surface chemistry of carbon materials. This method enables the quantification of surface-oxygenated functional groups. Calculated (based on calibrated gases) and measured TPD-MS pressure profiles are shown in Figure S6 (Supporting Information) for the pristine and 4.6 V samples. The pressure profiles for samples at 4.0, 4.2, and 4.4 V are similar to those for 4.6 V. The temperatures at which the two curves do not overlap show the presence of uncalibrated gases. These uncalibrated gases prevent accurate quantification of the calibrated gases at the temperature at which they have evolved due to the contribution of additional *m*/*z* to the total intensity of the *m*/*z* of the calibrated gases. From Figure S6, an uncalibrated peak at approximately 200 °C is present in both pristine and cycled electrodes, potentially due to the physisorbed contamination. Two additional uncalibrated peaks are observed at 230 and 360 °C for all cycled materials but are absent in the pristine material. In particular, the peak at 360 °C is very intense, indicating the presence of uncalibrated gases. To obtain more insight into the nature of the gases that are evolved in these regions, Figure 6 shows the MS profiles. Graphs a–e show the uncalibrated *m*/*z* species.

Several masses, which can be related to fluorinated functional groups or decomposition of the LiPF_6_ electrolyte, are recorded, such as those of COF, COF_2_, and CF_2_. The release of HF can also be observed, which is potentially responsible for the fluorination of the carbon structure and the release of fluorinated groups. HF can also lead to fluorination of the silica crucible, as indicated by the presence of mass *m*/*z* = 85; this is possibly the result of the decomposition of silica-fluorinated groups created during heat treatment with TPD-MS.^42^ Mass *m*/*z* = 85 can also be associated with a fragment peak of POF_3_, which produces a *m*/*z* = 104 (Figure S7). POF_3_ can be formed by the decomposition of carbonates and LiPF_6_ electrolytes.^47^ At 400 °C, *m*/*z* = 85 and 104 nm are simultaneously detected, showing the presence of POF_3_. In contrast, only *m*/*z* = 85 is present at 200 °C, which indicates the decomposition of silica-fluorinated groups at this temperature. In addition, *m*/*z* = 90 and 118 are correlated with dimethyl carbonate (DMC) and ethyl carbonate (EC), respectively; both are observed at 380 °C. These two electrolyte species can either be trapped in the porosity of the electrode or strongly bonded to the carbon surface, most likely due to electrolyte decomposition, and are not removed during the washing step. However, the measured intensities for the masses associated with fluorinated compounds, EC, and DMC are very low (3 orders of magnitude) compared to oxygen-based groups, as illustrated by the comparison with Figure 5e, which shows *m*/*z* = 28 (CO) and *m*/*z* = 44 (CO_2_) for sample 4.6 V. Although the TPD-MS results cannot be strictly compared with the XPS (due to their differences), one might note that XPS also shows low amounts of fluorine and phosphorus groups in the materials. Figure 5a,b shows the CO_2_ and CO desorption rate profiles of the five samples.

In the low-temperature region, two uncalibrated peaks are observed at 225 and 352 °C; thus, these uncalibrated species also decompose as *m*/*z* = 28 and 44. The peak of CO_2_ at this temperature is potentially due to the decomposition of carbonates, such as Li_2_CO_3_ and lithium alkyl carbonate species (ROCO_2_Li). The CO peak is possibly the result of the decomposition of short electrolyte polymer chains, such as the poly(ethylene oxide) polymer (CH_2_–CH_2_–O) formed on the electrode surface, via the polymerization of EC.^41,42^

Therefore, in this temperature range, the nature of the oxygenated surface groups created during cycling because of the contribution from the uncalibrated compounds is difficult to deduce. However, carboxylic and anhydride groups are susceptible and likely formed, leading to CO_2_ and CO groups.^48^ In the high-temperature region (between 650 and 950 °C), only calibrated gases evolve, as demonstrated by the two pressure curves that overlap above 600 °C. In this temperature range, the CO_2_ profiles have a low intensity and similar shapes (Figure 5a), while significant differences are observed in the CO profiles (Figure 5b). For voltages higher than 4.2 V, a new CO peak at ∼700 °C is observed and not present in the pristine and 4.0 V electrodes. This peak is attributed to ether and phenol surface groups,^49,50^ and its similar intensity for 4.2, 4.4, and 4.6 V electrodes indicate a similar amount. However, the CO peak at 874 °C, showing the presence of oxygenated surface groups, such as carbonyl and quinone, is present in both the pristine and cycled materials, and its intensity increases with voltage. Therefore, these oxygen functionalities continuously form with an increasing voltage. Figure 5c,d shows the total amount of CO_2_ and CO that evolved during the TPD-MS analysis, respectively, from room temperature to 950 °C and between 600 and 950 °C. The second temperature range is interesting because it provides accurate amounts of total CO_2_ and CO without the contribution of uncalibrated gases. Therefore, between 600 and 950 °C, the amounts of CO_2_ and CO are only due to oxygenated surface groups. Figure 5c also shows that the amount of carbonates, short electrolyte polymer chains, and oxygenated surface groups increases significantly and reaches a peak at 4.4 V. In Figure 5d, above 600 °C, the amount of CO_2_ is rather low, while there is an increase in the total amount of CO with voltage, with a peak at 4.4 V also.

These measurements demonstrated an increase in the oxygen-based functional groups with voltage, which is in good agreement with the elemental analysis results. Moreover, these results indicate the presence of by-products coming from the polymerization of solvents, carbonate formation, and, to a lesser degree, the fluorination of carbon. The presence of two strong CO and CO_2_ peaks that originated from the decomposition of Li_2_CO_3_ and polymer chains supports the theory of fast electrolyte decomposition under overcharged conditions. However, several general reasons for the system degradation of LiC can be distinguished.

### 3.1 Electrolyte Degradation

The interactions between the electrolyte and the electrode can promote the electrolyte decomposition with the formation of SEI and organic/inorganic by-products that will cover the carbon surface or block their porosity porosity and active sites. These undesired reactions can be favored at high voltage.

### 3.2 Carbon Property Modification

(i) Porosity (specific surface area and pore volume) changes are caused by pore clogging due to unsolvable species (from electrolyte degradation) and O functional groups, leading to fewer adsorbed species, slower diffusion, and lower capacitance.^9^
(ii) Surface chemistry and oxidation of carbon surface are changed, leading to lower conductivity.^51^ The oxygen-based functional groups can disrupt the hexagonal sp^2^-bonded carbon network, diminishing the electron mobility and electrical conductivity. They can also participate in undesired reactions with the electrolyte at high voltages.
(iii) Structural changes (defects coming from C sp^3^, C–O bonds, polymerized monomers) contribute to the electrolyte decomposition and increase resistance (decreased conductivity).

### 3.3 Voltage Applied

The increase in voltage might induce faster electrolyte and electrode degradation, as well as gas generation and pressure increase.^52^ Also, as the time of the experiment was prolonged, the changes in pore volume in consecutive floating cycles resulted in structure degradation possibly connected with pore clogging. However, at higher voltage, faster oxidation and functionalization of carbon lead to an increase in resistance, resulting in less affected pore structure.

Additionally, one needs to remember that the formation of the SEI layer on the counter electrode might also contribute to the increase in resistance.

**Figure 7:**
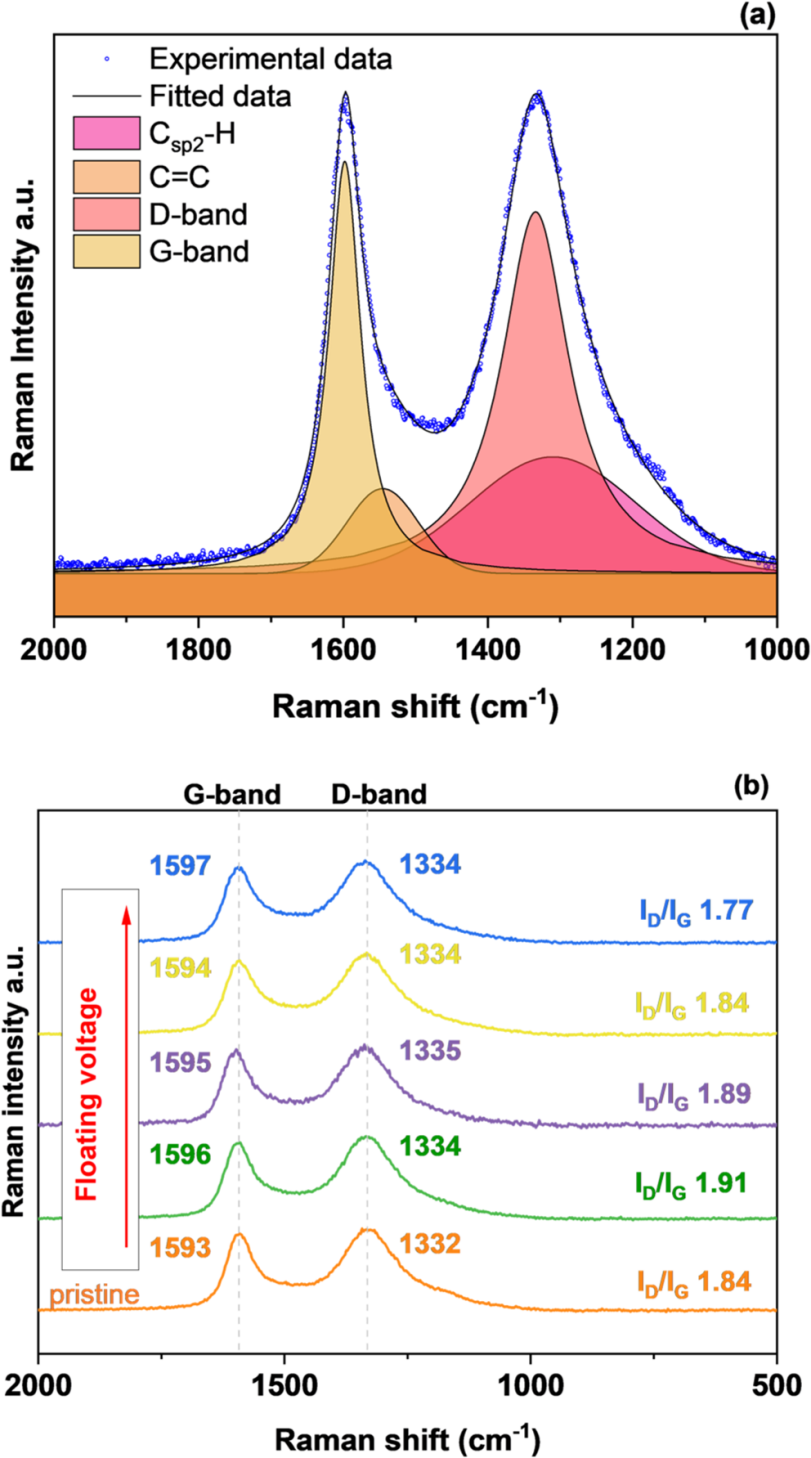
Raman spectroscopy analysis of the positive electrodes after the floating test at various voltages from 4.0 to 4.6 V compared to that of the pristine carbon cloth before aging: (a) deconvoluted spectrum of the pristine Kynol 507-20 electrode and (b) Raman spectrum of the electrodes with the marked positions of the peaks and *I*_D_/*I*_G_ ratio.

Structural changes were monitored also by Raman spectroscopy and the D/G band intensity ratio calculations from spectra deconvolution.^53,54^ The experimental data and fitted curves of the pristine Kynol 507–20 carbon are shown in Figure 7a.

The ratio of the intensity of the D/G bands is a measure of the defects present in the graphene structure. The G band is the result of in-plane vibrations of sp^2^-bonded carbon atoms, while the D band is due to the out-of-plane vibrations attributed to the presence of structural defects. The D mode is present in all graphite-like carbons and originates from structural defects. A comparison of the *I*_D_/*I*_G_ ratio calculated from the integrated area of the deconvoluted peaks presented in Figure 7b shows an insignificant increase in the D/G peak intensity ratio; however, the effect diminished with the increasing voltage.

The lack of significant changes in the *I*_D_/*I*_G_ ratio can potentially result from low laser beam penetration. As observed by XPS, much of the oxygen content on the surface of carbon comes from the products of electrolyte decomposition in the form of polymer-like carbonates and Li_2_CO_3_.^28^ However, as observed by TPD-MS and EA, carbon bulk oxidation clearly takes place, which cannot be observed by Raman spectroscopy. Furthermore, the D and G bands remain almost constant for all materials (Figure 7b).

## 4 Conclusions

The electrochemical aging of the carbon electrode in LiC, followed by a physiochemical analysis, was presented and discussed. Results from elemental analysis, porosimetry, mass spectrometry, XPS, and Raman spectroscopy provided a complete picture of the capacitance/energy fading during the accelerated aging test. Therefore, several observations and conclusions could be drawn:

- Depending on the maximum voltage, the systems reached different end-of-life criteria: (i) at 4.0 V: capacitance fades with simultaneous resistance increase; and (ii) between 4.2 and 4.6 V: a similar system failure reason occurred with more pronounced resistance increase;
  aging at 4.0 V: chemical and structural changes of the electrode were observed. Combining the data from N_2_ adsorption and TPD-MS enabled the existence of a spatial hindrance in the form of residuals of electrolyte decomposition resulting in pore clogging and specific surface area decrease.
- Aging voltage higher than 4.2 V: The formation of ether and phenol groups was confirmed, resulting in the resistance increase.^42^ Further observations via EA, TPD-MS, and XPS confirm the increase in the overall oxygen content. This has two different origins. First, additional sources of oxygen (as identified via XPS) were found at the surface of the electrode coming from electrolyte decomposition in the form of poly(ethylene oxides), polymer-like carbonates, and Li_2_CO_3_. The amount/nature of these species might vary with voltage and impact their localization in the carbon (micro- or mesopores), with a direct consequence on textural properties. Second, carbon oxidation in the bulk of the material leads to enhanced oxygen content, as recorded via TPD-MS and EA. These results confirm that higher aging voltage induces faster oxidation and carbon functionalization. The formation of new O-functionalities led to a decrease in the conductive C sp^2^ content, and their insulating nature contributed to an increase in the resistance and degradation of capacitor performance. Additionally, the potential corrosion of the system caused by the presence of HF needed to be considered and could lead to an increase in cell resistance.^40,55^

Our data show that in the case of LiCs, different aging pathways for the carbon electrode need to be considered during the optimization of the full cell.

## Supporting Information Available

Operando pressure measurement during charge/discharge recorded after the first seven floating sequences at 4 V; charge/discharge cycles before and after floating; pore size distribution for pristine carbon cloth and the positive electrodes after the floating tests; elemental analysis of the pristine and aged positive electrodes; deconvoluted XPS C 1s spectra for pristine and aged carbon electrode after aging at 4.0 and 4.6 V; TPD-MS pressure profiles for pristine and 4.6 V electrodes; theoretical MS spectra from NIST MS search database of dimethyl carbonate; and diethyl carbonate, CF_4_, and POF_3_ (PDF)

## References

[ref1] Yu S. J.; Ng V. M. H.; Wang F. J.; Xiao Z. H.; Li C. Y.; Kong L. B.; Que W. X.; Zhou K. Synthesis and application of iron-based nanomaterials as anodes of lithium-ion batteries and supercapacitors. J. Mater. Chem. A 2018, 6 (20), 9332–9367. 10.1039/c8ta01683f.

[ref2] Simon P.; Gogotsi Y. Materials for electrochemical capacitors. Nat. Mater. 2008, 7 (11), 845–854. 10.1038/nmat2297. 18956000

[ref3] Winter M.; Brodd R. J. What are batteries, fuel cells, and supercapacitors?. Chem. Rev. 2004, 104 (10), 4245–4269. 10.1021/cr020730k. 15669155

[ref4] Abruña H. D.; Kiya Y.; Henderson J. C. Batteries and electrochemical capacitors. Phys. Today 2008, 61 (12), 43–47. 10.1063/1.3047681.

[ref5] The Top Ten Scientific Questions in Electrochemistry. J. Electrochem. 2024, 30 (1), 2024121 10.61558/2993-074X.3444.

[ref6] Simon P.; Gogotsi Y. Perspectives for electrochemical capacitors and related devices. Nat. Mater. 2020, 19 (11), 1151–1163. 10.1038/s41563-020-0747-z. 32747700

[ref7] Lewandowski A.; Galinski M. Practical and theoretical limits for electrochemical double-layer capacitors. J. Power Sources 2007, 173 (2), 822–828. 10.1016/j.jpowsour.2007.05.062.

[ref8] González A.; Goikolea E.; Barrena J. A.; Mysyk R. Review on supercapacitors: Technologies and materials. Renewable Sustainable Energy Rev. 2016, 58, 1189–1206. 10.1016/j.rser.2015.12.249.

[ref9] El Ghossein N.; Sari A.; Venet P.; Genies S.; Azaïs P. Post-Mortem Analysis of Lithium-Ion Capacitors after Accelerated Aging Tests. J. Energy Storage 2021, 33, 102039 10.1016/j.est.2020.102039.

[ref10] Gogotsi Y.; Simon P. True performance metrics in electrochemical energy storage. Science 2011, 334 (6058), 917–918. 10.1126/science.1213003. 22096182

[ref11] Sun X. Z.; Zhang X.; Liu W. J.; Wang K.; Li C.; Li Z.; Ma Y. W. Electrochemical performances and capacity fading behaviors of activated carbon/hard carbon lithium ion capacitor. Electrochim. Acta 2017, 235, 158–166. 10.1016/j.electacta.2017.03.110.

[ref12] Naoi K.; Ishimoto S.; Miyamoto J.; Naoi W. Second generation ’nanohybrid supercapacitor’: Evolution of capacitive energy storage devices. Energy Environ. Sci. 2012, 5 (11), 9363–9373. 10.1039/c2ee21675b.

[ref13] Dsoke S.; Fuchs B.; Gucciardi E.; Wohlfahrt-Mehrens M. The importance of the electrode mass ratio in a Li-ion capacitor based on activated carbon and Li_4_Ti_5_O_12_. J. Power Sources 2015, 282, 385–393. 10.1016/j.jpowsour.2015.02.079.

[ref14] Cao W. J.; Shih J.; Zheng J. P.; Doung T. Development and characterization of Li-ion capacitor pouch cells. J. Power Sources 2014, 257, 388–393. 10.1016/j.jpowsour.2014.01.087.

[ref15] Zhang J.; Liu X. F.; Wang J.; Shi J. L.; Shi Z. Q. Different types of pre-lithiated hard carbon as negative electrode material for lithium-ion capacitors. Electrochim. Acta 2016, 187, 134–142. 10.1016/j.electacta.2015.11.055.

[ref16] Keil P.; Schuster S. F.; Wilhelm J.; Travi J.; Hauser A.; Karl R. C.; Jossen A. Calendar Aging of Lithium-Ion Batteries I. Impact of the Graphite Anode on Capacity Fade. J. Electrochem. Soc. 2016, 163 (9), A1872–A1880. 10.1149/2.0411609jes.

[ref17] Aida T.; Murayama I.; Yamada K.; Morita M. Analyses of capacity loss and improvement of cycle performance for a high-voltage hybrid electrochemical capacitor. J. Electrochem. Soc. 2007, 154 (8), A798–A804. 10.1149/1.2746562.

[ref18] Ruch P. W.; Cericola D.; Foelske-Schmitz A.; Kötz R.; Wokaun A. Aging of electrochemical double layer capacitors with acetonitrile-based electrolyte at elevated voltages. Electrochim. Acta 2010, 55 (15), 4412–4420. 10.1016/j.electacta.2010.02.064.

[ref19] Weingarth D.; Noh H.; Foelske-Schmitz A.; Wokaun A.; Kötz R. A reliable determination method of stability limits for electrochemical double layer capacitors. Electrochim. Acta 2013, 103, 119–124. 10.1016/j.electacta.2013.04.057.

[ref20] Oukaour A.; Tala-Ighil B.; AlSakka M.; Gualous H.; Gallay R.; Boudart B. Calendar ageing and health diagnosis of supercapacitor. Electr. Power Syst. Res. 2013, 95, 330–338. 10.1016/j.epsr.2012.09.005.

[ref21] Liu Y. H.; Réty B.; Ghimbeu C. M.; Soucaze-Guillous B.; Taberna P. L.; Simon P. Understanding ageing mechanisms of porous carbons in non-aqueous electrolytes for supercapacitors applications. J. Power Sources 2019, 434, 226734 10.1016/j.jpowsour.2019.226734.

[ref22] Bittner A. M.; Zhu M.; Yang Y.; Waibel H. F.; Konuma M.; Starke U.; Weber C. J. Ageing of electrochemical double layer capacitors. J. Power Sources 2012, 203, 262–273. 10.1016/j.jpowsour.2011.10.083.

[ref23] Cao W. J.; Zheng J. P. Li-ion capacitors with carbon cathode and hard carbon/stabilized lithium metal powder anode electrodes. J. Power Sources 2012, 213, 180–185. 10.1016/j.jpowsour.2012.04.033.

[ref24] Cao W. J.; Li Y. X.; Fitch B.; Shih J.; Doung T.; Zheng J. Strategies to optimize lithium-ion supercapacitors achieving high-performance: Cathode configurations, lithium loadings on anode, and types of separator. J. Power Sources 2014, 268, 841–847. 10.1016/j.jpowsour.2014.06.090.

[ref25] Sun X.; Zhang X.; Wang K.; An Y.; Zhang X.; Li C.; Ma Y. Determination strategy of stable electrochemical operating voltage window for practical lithium-ion capacitors. Electrochim. Acta 2022, 428, 140972 10.1016/j.electacta.2022.140972.

[ref26] Song S.; Zhang X.; Li C.; Wang K.; Sun X.; Ma Y. Anomalous diffusion models in frequency-domain characterization of lithium-ion capacitors. J. Power Sources 2021, 490, 229332 10.1016/j.jpowsour.2020.229332.

[ref27] Babu B.; Neumann C.; Enke M.; Lex-Balducci A.; Turchanin A.; Schubert U. S.; Balducci A. Aging processes in high voltage lithium-ion capacitors containing liquid and gel-polymer electrolytes. J. Power Sources 2021, 496, 229797 10.1016/j.jpowsour.2021.229797.

[ref28] Piwek J.; Platek A.; Frackowiak E.; Fic K. Mechanisms of the performance fading of carbon-based electrochemical capacitors operating in a LiNO3 electrolyte. J. Power Sources 2019, 438, 227029 10.1016/j.jpowsour.2019.227029.

[ref29] Platek A.; Piwek J.; Fic K.; Frackowiak E. Ageing mechanisms in electrochemical capacitors with aqueous redox-active electrolytes. Electrochim. Acta 2019, 311, 211–220. 10.1016/j.electacta.2019.04.117.

[ref30] Zallouz S.; Le Meins J. M.; Ghimbeu C. M. Alkaline hydrogel electrolyte from biosourced chitosan to enhance the rate capability and energy density of carbon-based supercapacitors. Energy Adv. 2022, 1 (12), 1051–1064. 10.1039/d2ya00250g.

[ref31] Balducci A.; Belanger D.; Brousse T.; Long J. W.; Sugimoto W. Perspective—A Guideline for Reporting Performance Metrics with Electrochemical Capacitors: From Electrode Materials to Full Devices. J. Electrochem. Soc. 2017, 164 (7), A1487–A1488. 10.1149/2.0851707jes.

[ref32] Weingarth D.; Foelske-Schmitz A.; Kötz R. Cycle versus voltage hold – Which is the better stability test for electrochemical double layer capacitors?. J. Power Sources 2013, 225, 84–88. 10.1016/j.jpowsour.2012.10.019.

[ref33] Zhao J.; Burke A. F. Review on supercapacitors: Technologies and performance evaluation. J. Energy Chem. 2021, 59, 276–291. 10.1016/j.jechem.2020.11.013.

[ref34] Rizoug N.; Bartholomeus P.; Moigne P. L. Study of the Ageing Process of a Supercapacitor Module Using Direct Method of Characterization. IEEE Trans. Energy Convers. 2012, 27 (2), 220–228. 10.1109/TEC.2012.2186814.

[ref35] Zhao L.; Watanabe I.; Doi T.; Okada S.; Yamaki J.-i. TG-MS analysis of solid electrolyte interphase (SEI) on graphite negative-electrode in lithium-ion batteries. J. Power Sources 2006, 161 (2), 1275–1280. 10.1016/j.jpowsour.2006.05.045.

[ref36] Jagiello J.; Olivier J. P. 2D-NLDFT adsorption models for carbon slit-shaped pores with surface energetical heterogeneity and geometrical corrugation. Carbon 2013, 55, 70–80. 10.1016/j.carbon.2012.12.011.

[ref37] Jagiello J.; Olivier J. P. Carbon slit pore model incorporating surface energetical heterogeneity and geometrical corrugation. Adsorption 2013, 19 (2–4), 777–783. 10.1007/s10450-013-9517-4.

[ref38] Ghimbeu C. M.; Gadiou R.; Dentzer J.; Schwartz D.; Vix-Guterl C. Influence of surface chemistry on the adsorption of oxygenated hydrocarbons on activated carbons. Langmuir 2010, 26 (24), 18824–18833. 10.1021/la103405j. 21117633

[ref39] Lou S.; Liu Q.; Zhang F.; Liu Q.; Yu Z.; Mu T.; Zhao Y.; Borovilas J.; Chen Y.; Ge M.; Xiao X.; Lee W.; Yin G.; Yang Y.; Sun X.; Wang J. Insights into interfacial effect and local lithium-ion transport in polycrystalline cathodes of solid-state batteries. Nat. Commun. 2020, 11 (1), 5700 10.1038/s41467-020-19528-9. 33177510 PMC7658997

[ref40] Bolli C.; Guéguen A.; Mendez M. A.; Berg E. J. Operando Monitoring of F-Formation in Lithium Ion Batteries. Chem. Mater. 2019, 31 (4), 1258–1267. 10.1021/acs.chemmater.8b03810.

[ref41] Matei Ghimbeu C.; Vidal L.; Delmotte L.; Le Meins J.-M.; Vix-Guterl C. Catalyst-free soft-template synthesis of ordered mesoporous carbon tailored using phloroglucinol/glyoxylic acid environmentally friendly precursors. Green Chem. 2014, 16 (6), 3079–3088. 10.1039/c4gc00269e.

[ref42] Matei Ghimbeu C.; Guerin K.; Dubois M.; Hajjar-Garreau S.; Vix-Guterl C. Insights on the reactivity of ordered porous carbons exposed to different fluorinating agents and conditions. Carbon 2015, 84, 567–583. 10.1016/j.carbon.2014.12.034.

[ref43] Dedryvère R.; Gireaud L.; Grugeon S.; Laruelle S.; Tarascon J. M.; Gonbeau D. Characterization of lithium alkyl carbonatesby X-ray photoelectron spectroscopy: experimental and theoretical study. J. Phys. Chem. B 2005, 109 (33), 15868–15875. 10.1021/jp051626k. 16853016

[ref44] Peled E.; Menkin S. Review—SEI: Past, Present and Future. J. Electrochem. Soc. 2017, 164 (7), A1703 10.1149/2.1441707jes.

[ref45] Adenusi H.; Chass G. A.; Passerini S.; Tian K. V.; Chen G. Lithium Batteries and the Solid Electrolyte Interphase (SEI)—Progress and Outlook. Adv. Energy Mater. 2023, 13 (10), 2203307 10.1002/aenm.202203307.

[ref46] Li J.; Ji Y.; Song H.; Chen S.; Ding S.; Zhang B.; Yang L.; Song Y.; Pan F. Insights Into the Interfacial Degradation of High-Voltage All-Solid-State Lithium Batteries. Nano-Micro Lett. 2022, 14 (1), 191 10.1007/s40820-022-00936-z. PMC9485319 36121521

[ref47] Guéguen A.; Streich D.; He M.; Mendez M.; Chesneau F. F.; Novák P.; Berg E. J. Decomposition of LiPF6in High Energy Lithium-Ion Batteries Studied with Online Electrochemical Mass Spectrometry. J. Electrochem. Soc. 2016, 163 (6), A1095–A1100. 10.1149/2.0981606jes.

[ref48] Figueiredo J. L.; Pereira M. F. R.; Freitas M. M. A.; Orfao J. J. M. Modification of the surface chemistry of activated carbons. Carbon 1999, 37 (9), 1379–1389. 10.1016/S0008-6223(98)00333-9.

[ref49] Brender P.; Gadiou R.; Rietsch J. C.; Fioux P.; Dentzer J.; Ponche A.; Vix-Guterl C. Characterization of carbon surface chemistry by combined temperature programmed desorption with in situ X-ray photoelectron spectrometry and temperature programmed desorption with mass spectrometry analysis. Anal. Chem. 2012, 84 (5), 2147–2153. 10.1021/ac102244b. 22242697

[ref50] Moussa G.; Ghimbeu C. M.; Taberna P. L.; Simon P.; Vix-Guterl C. Relationship between the carbon nano-onions (CNOs) surface chemistry/defects and their capacitance in aqueous and organic electrolytes. Carbon 2016, 105, 628–637. 10.1016/j.carbon.2016.05.010.

[ref51] Eleri O. E.; Azuatalam K. U.; Minde M. W.; Trindade A. M.; Muthuswamy N.; Lou F.; Yu Z. Towards high-energy-density supercapacitors via less-defects activated carbon from sawdust. Electrochim. Acta 2020, 362, 137152 10.1016/j.electacta.2020.137152.

[ref52] Edge J. S.; O’Kane S.; Prosser R.; Kirkaldy N. D.; Patel A. N.; Hales A.; Ghosh A.; Ai W.; Chen J.; Yang J.; Li S.; Pang M.; Diaz L. B.; Tomaszewska A.; Marzook M. W.; Radhakrishnan K. N.; Wang H.; Patel Y.; Wu B.; Offer G. J. Lithium ion battery degradation: what you need to know. Phys. Chem. Chem. Phys. 2021, 23 (14), 8200–8221. 10.1039/D1CP00359C. 33875989

[ref53] Couzi M.; Bruneel J. L.; Talaga D.; Bokobza L. A multi wavelength Raman scattering study of defective graphitic carbon materials: The first order Raman spectra revisited. Carbon 2016, 107, 388–394. 10.1016/j.carbon.2016.06.017.

[ref54] Bokobza L.; Bruneel J. L.; Couzi M. Raman Spectra of Carbon-Based Materials (from Graphite to Carbon Black) and of Some Silicone Composites. C 2015, 1 (1), 77–94. 10.3390/c1010077.

[ref55] Lux S. F.; Lucas I. T.; Pollak E.; Passerini S.; Winter M.; Kostecki R. The mechanism of HF formation in LiPF6 based organic carbonate electrolytes. Electrochem. Commun. 2012, 14 (1), 47–50. 10.1016/j.elecom.2011.10.026.

