# Supplementary material for: Identifying the Activated Carbon Electrode Aging Pathways in Lithium-Ion Hybrid Capacitors
Source: ACS Appl Energy Mater. 2025 Jan 10;8(2):810–20. doi: 10.1021/acsaem.4c01940 (PMC11775879; doi:10.1021/acsaem.4c01940)
Supplement: Supplementary file 1 — ae4c01940_si_001.pdf [file ae4c01940_si_001.pdf]

**Supporting Information**

for

# Identifying the activated carbon electrode ageing pathways in lithium-ion hybrid capacitors

*Sylwia Slesinska[1], Bénédicte Réty[2,3,4], Camélia Matei-Ghimbeu[2,3,4*], Krzysztof Fic[1], Jakub Menzel[1*]*

[1] Institute of Chemistry and Technical Electrochemistry, Poznan University of Technology, Berdychowo 4, 60-965 Poznan, Poland
[2] Institut de Science des Matériaux de Mulhouse (IS2M), Université de Haute-Alsace, CNRS UMR 7361, F-68100 Mulhouse, France;
[3]Université de Strasbourg, F67081 Strasbourg, France;
[4]Réseau sur le Stockage Electrochimique de l'Energie (RS2E), CNRS FR3459, 80039 Amiens Cedex, France

***Corresponding authors**
jakub.menzel@put.poznan.pl, camelia.ghimbeu@uha.fr

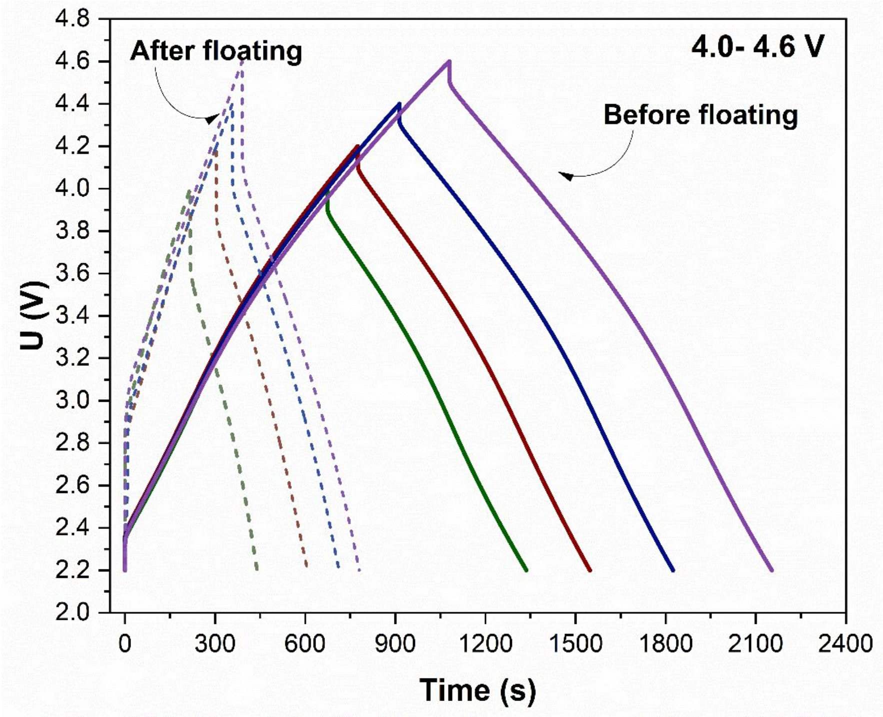


Figure S1 Charge/discharge curves before and after floating for systems 4.0- 4.6 V.

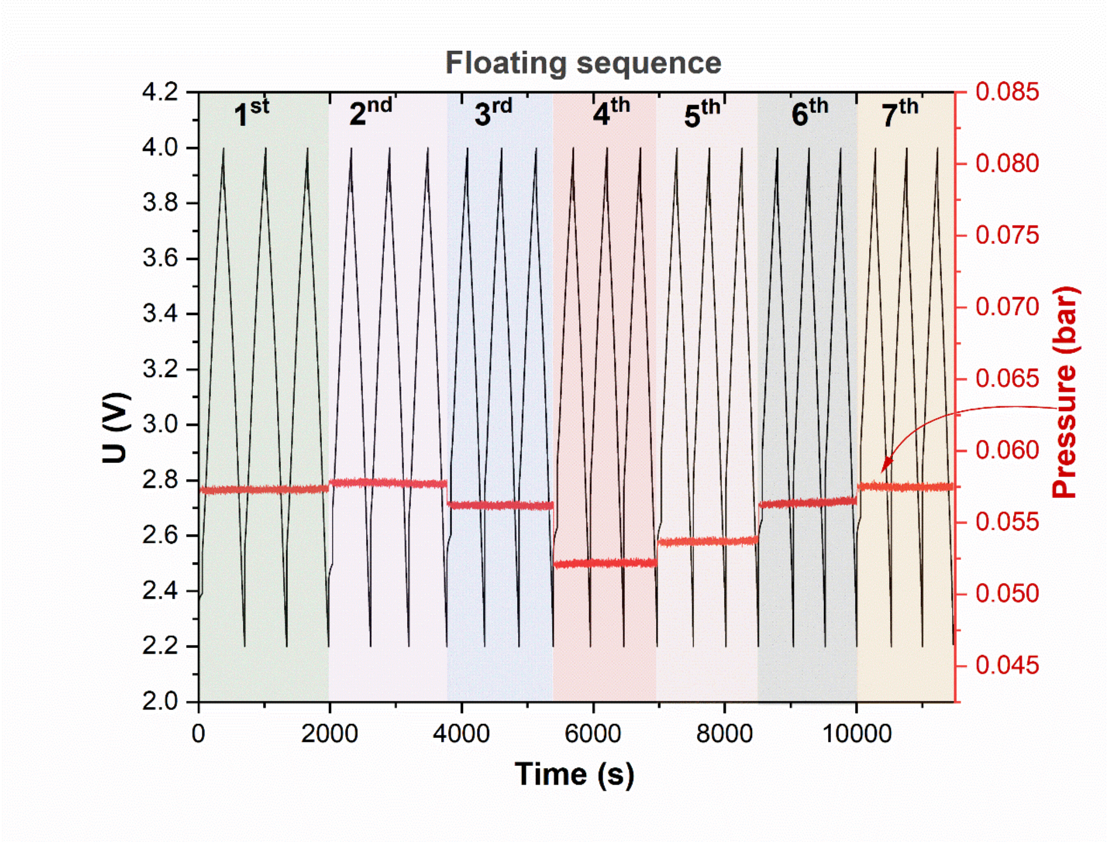


Figure S2 Operando pressure measurement during charge/discharge recorded after the first 7 floating sequences at 4V.

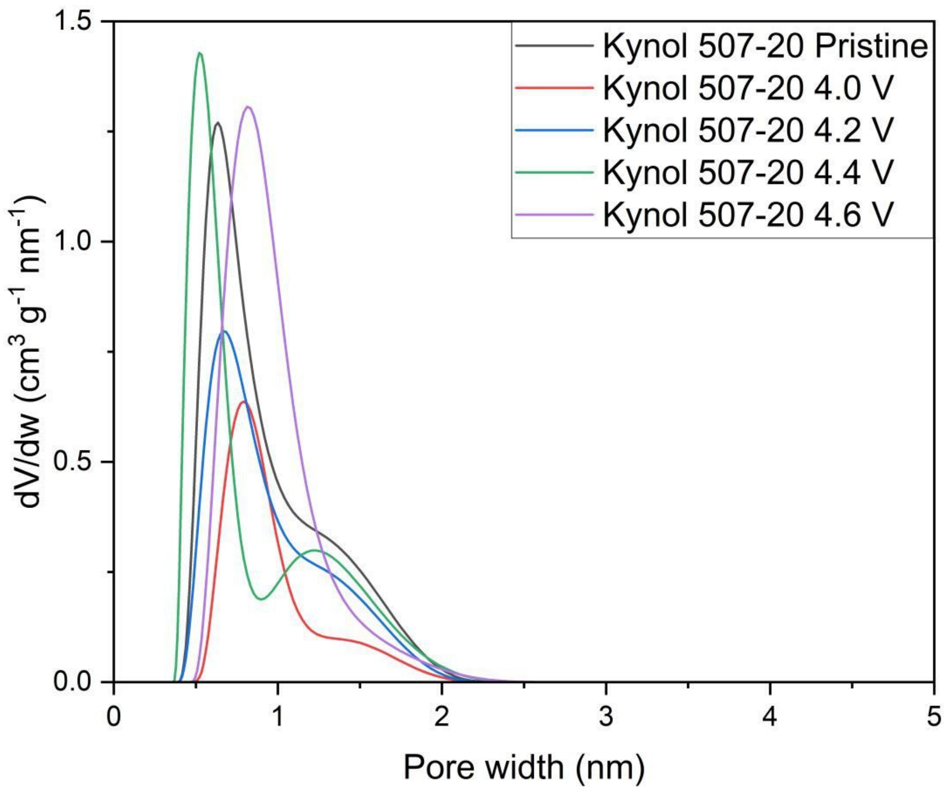


Figure S3 Pore size distribution for pristine carbon cloth and the positive electrodes after the floating test at various voltages (4V – 4.6V)

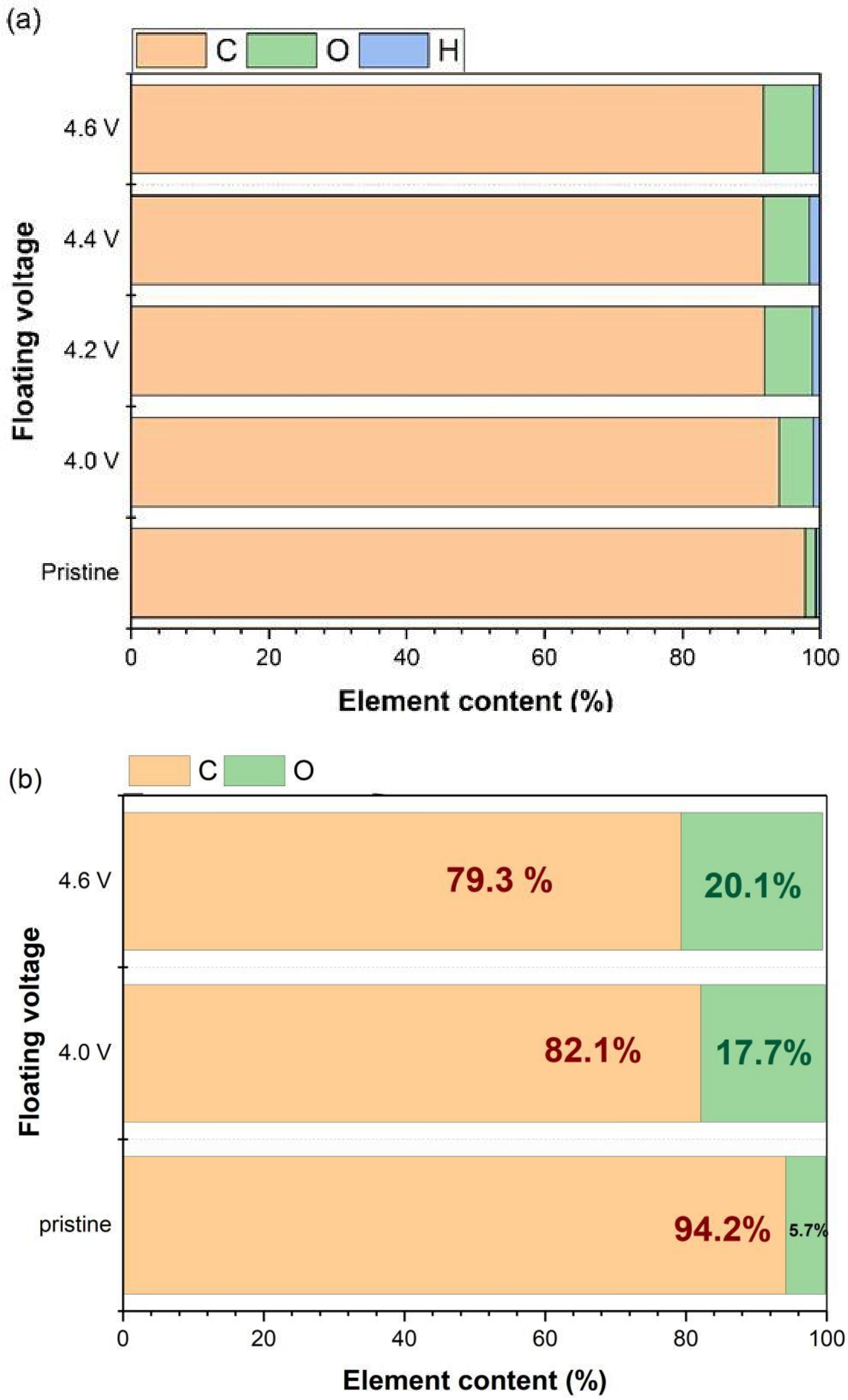


Figure S4 Elemental compositions of the positive electrodes after the floating test at various voltages compared to that of the carbon cloth before ageing, (a) from elemental analysis at 4.0– 4.6 V , (b) from XPS at 4.0 and 4.6 V.

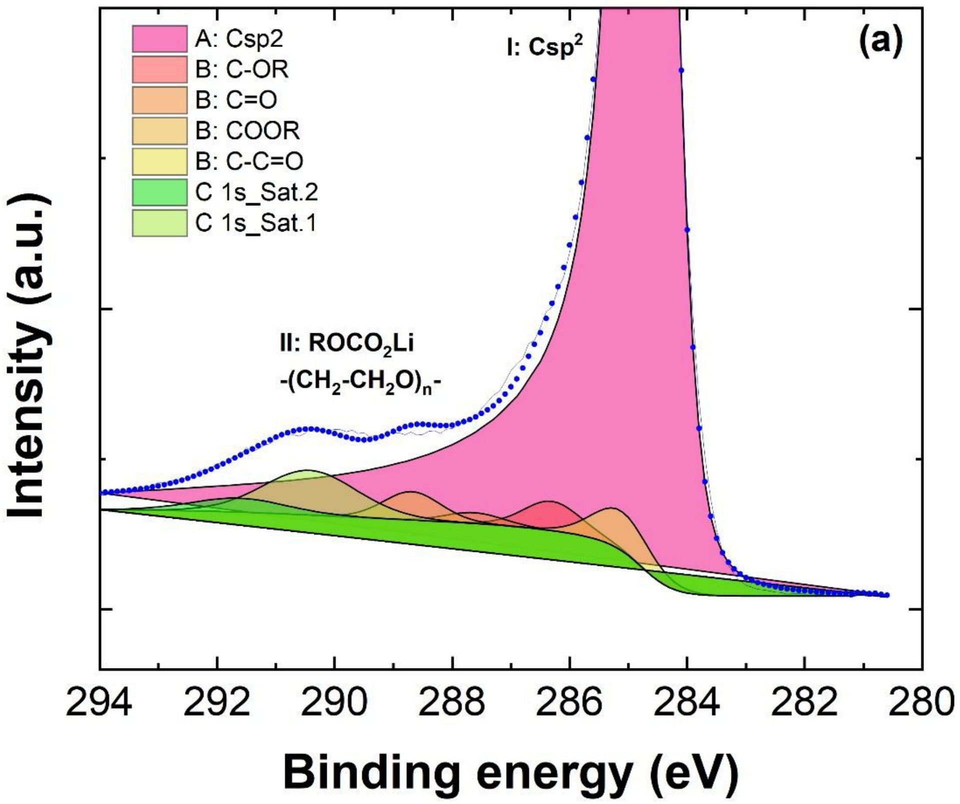

A: Csp2
B: C-OR
B: C=O
B: COOR
B: C-C=O
C 1s_Sat.2
C 1s_Sat.1
I: $Csp^2$
(a)
II: $ROCO_2Li$
$-(CH_2-CH_2O)_n-$
Intensity (a.u.)
294
292
290
288
286
284
282
280
Binding energy (eV)


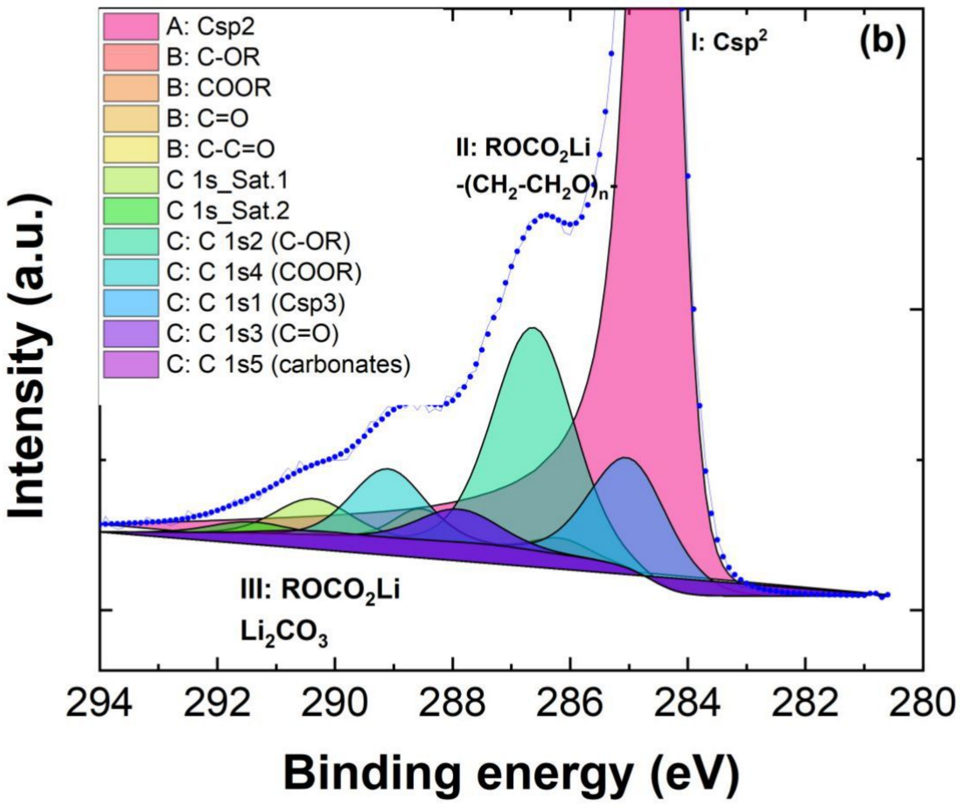

A: Csp2
B: C-OR
B: COOR
B: C=O
B: C-C=O
C 1s_Sat.1
C 1s_Sat.2
C: C 1s2 (C-OR)
C: C 1s4 (COOR)
C: C 1s1 (Csp3)
C: C 1s3 (C=O)
C: C 1s5 (carbonates)
I: $Csp^2$
(b)
II: $ROCO_2Li$
$-(CH_2-CH_2O)_n$
III: $ROCO_2Li$
$Li_2CO_3$
Intensity (a.u.)
294
292
290
288
286
284
282
280
Binding energy (eV)

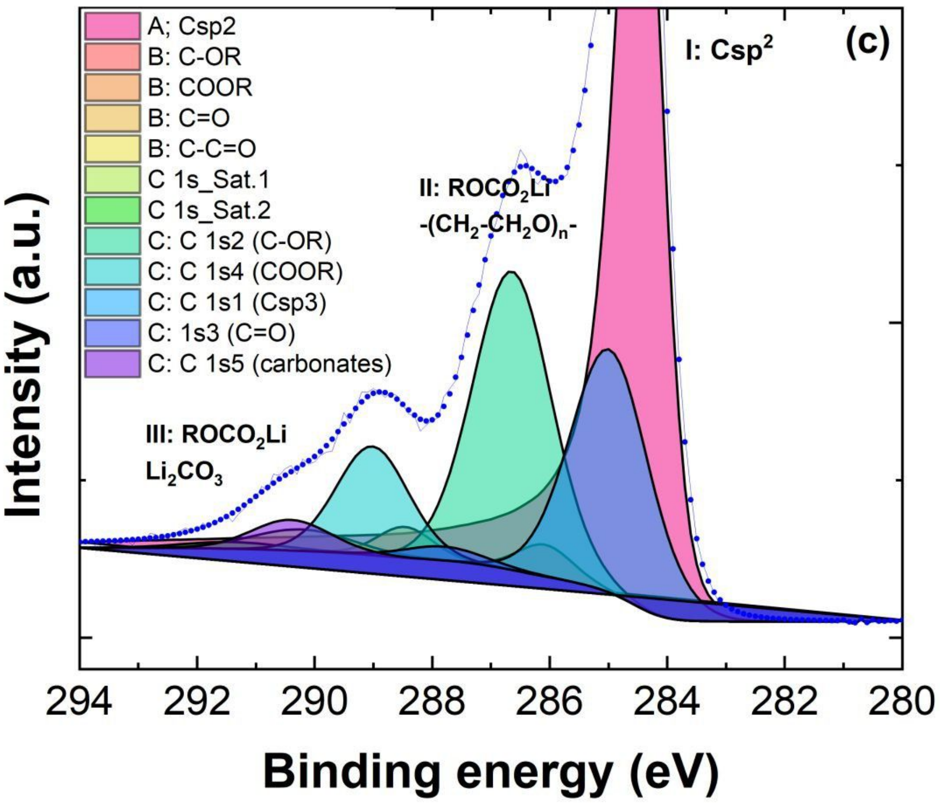


Figure S5 Deconvoluted XPS C1s spectra for a) pristine carbon; b) carbon electrode after aging at 4.0 V; c) carbon electrode after aging at 4.6 V; I corresponds to C sp$^2$, II corresponds to C-OR, COOR, C=O, and C-C=O bonded to C sp$^2$, and III corresponds to C-OR, COOR, C=O, C sp$^3$, and carbonate fractions

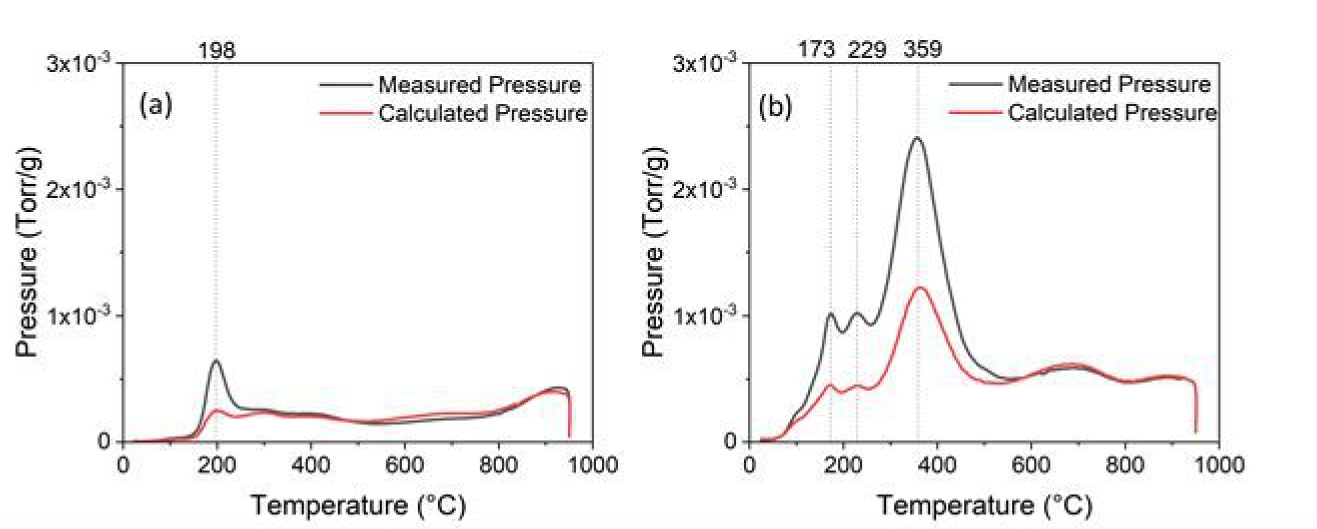


Figure S6 TPD-MS Pressure profiles (a) Pristine and (b) 4.6V electrodes

(a)

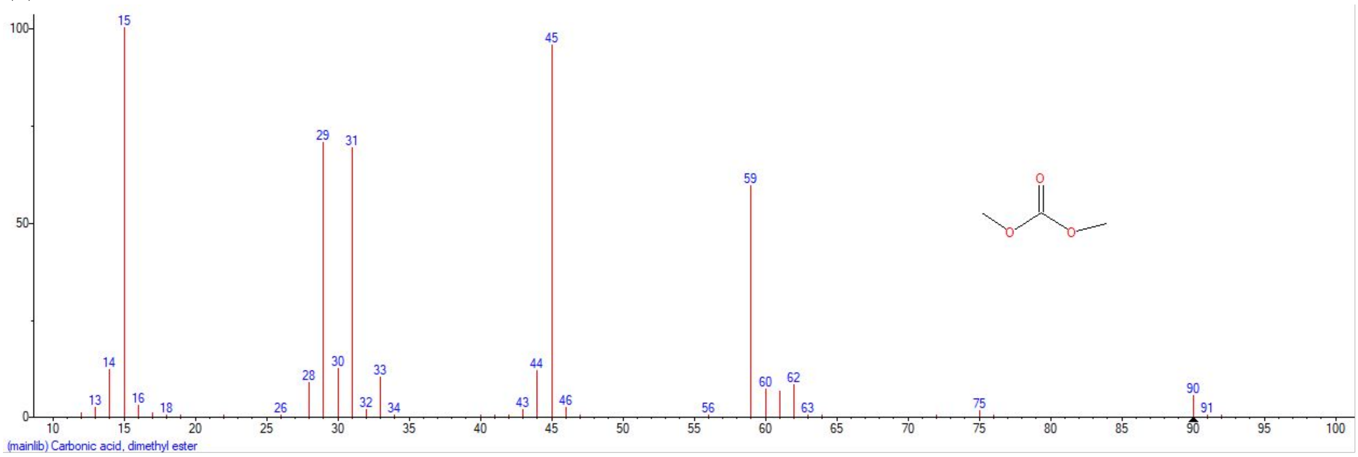


(b)

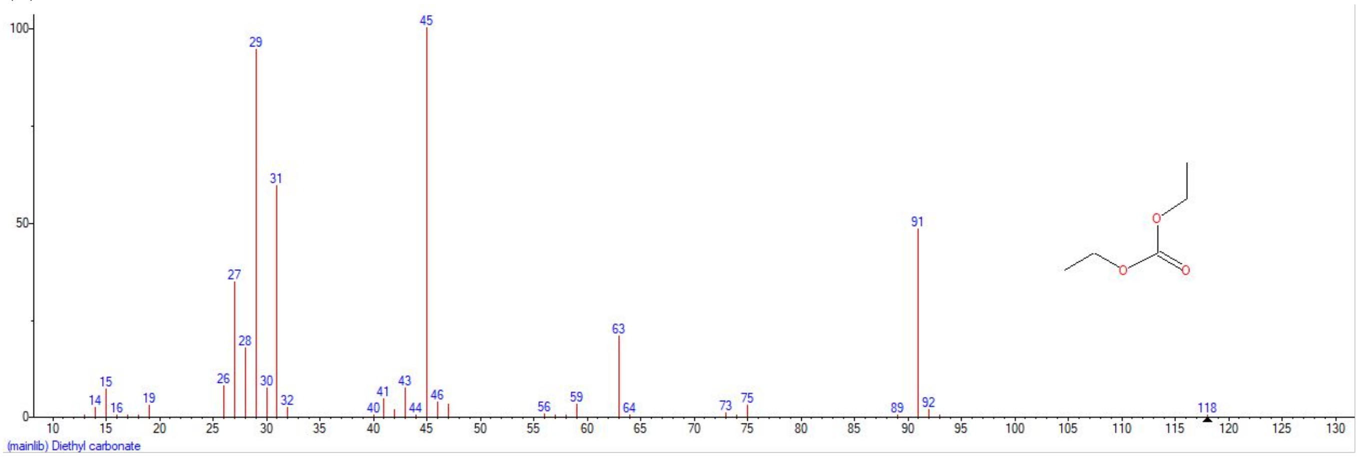


(c)

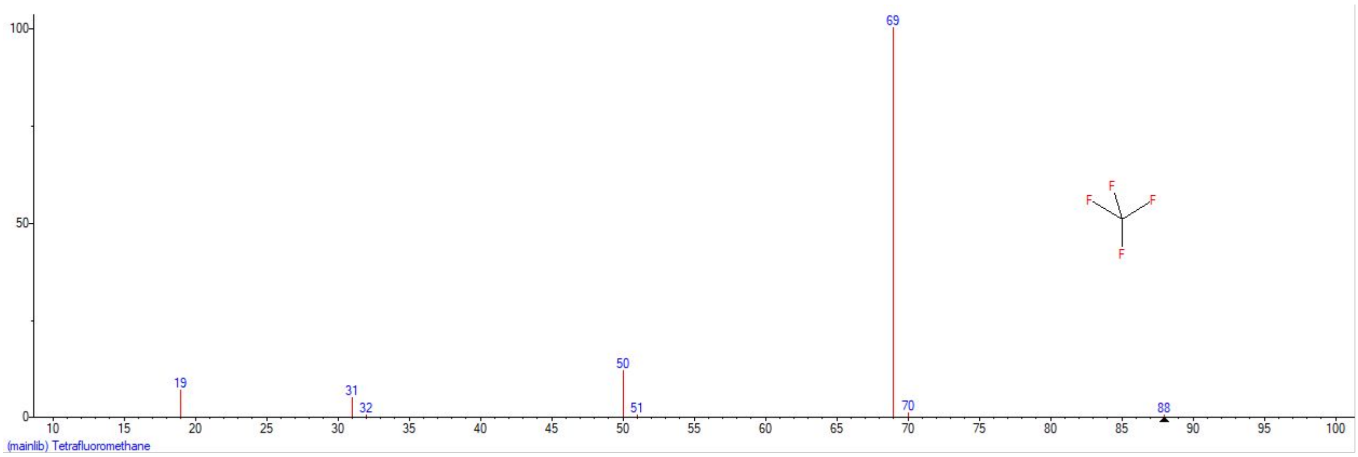


(d)

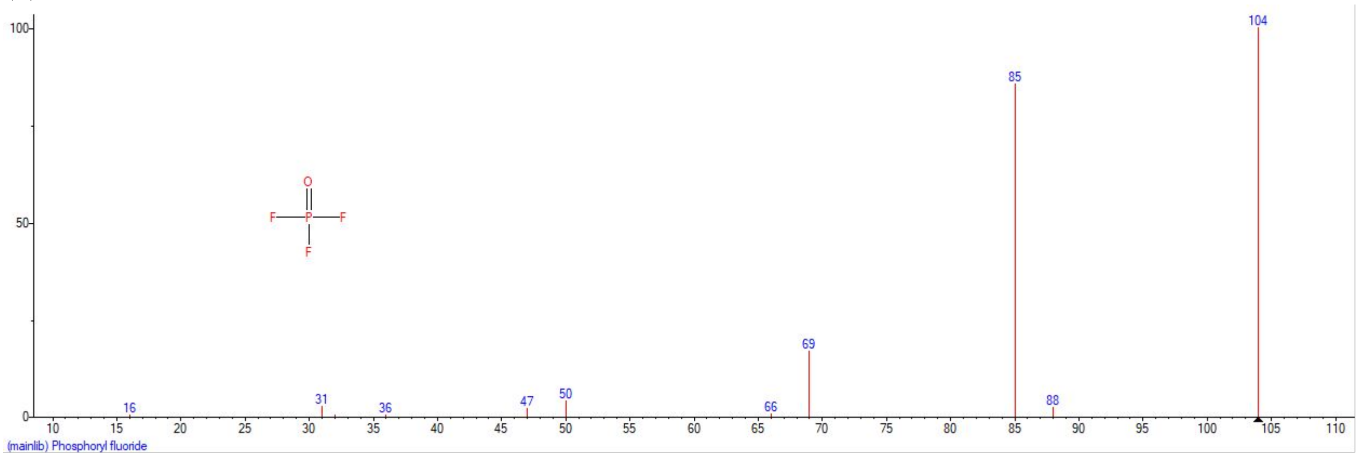


Figure S7 - Theoretical MS spectra from NIST MS Search data base (a) Dimethyl carbonate, (b) Diethyl carbonate, (c) $CF_4$, (d) $POF_3$
